# Automated quantification of *Candida albicans* biofilm-related phenotypes reveals additive contributions to biofilm production

**DOI:** 10.1038/s41522-020-00149-5

**Published:** 2020-10-09

**Authors:** Matthew J. Dunn, Robert J. Fillinger, Leah M. Anderson, Matthew Z. Anderson

**Affiliations:** 1 Department of Microbiology, The Ohio State University, Columbus, OH 43210 USA; 2 Biomedical Sciences Graduate Program, The Ohio State University, Columbus, OH 43210 USA; 3 Department of Microbial Infection and Immunity, The Ohio State University, Columbus, OH 43210 USA

**Keywords:** Biofilms, Pathogens, Biological techniques

## Abstract

Biofilms are organized communities of microbial cells that promote persistence among bacterial and fungal species. Biofilm formation by host-associated *Candida* species of fungi occurs on both tissue surfaces and implanted devices, contributing to host colonization and disease. In *C. albicans*, biofilms are built sequentially by adherence of yeast to a surface, invasion into the substrate, the formation of aerial hyphal projections, and the secretion of extracellular matrix. Measurement of these biofilm-related phenotypes remains highly qualitative and often subjective. Here, we designed an informatics pipeline for quantifying filamentation, adhesion, and invasion of *Candida* species on solid agar media and utilized this approach to determine the importance of these component phenotypes to *C. albicans* biofilm production. Characterization of 23 *C. albicans* clinical isolates across three media and two temperatures revealed a wide range of phenotypic responses among isolates in any single condition. Media profoundly altered all biofilm-related phenotypes among these isolates, whereas temperature minimally impacted these traits. Importantly, the extent of biofilm formation correlated significantly with the additive score for its component phenotypes under some conditions, experimentally linking the strength of each component to biofilm mass. In addition, the response of the genome reference strain, SC5314, across these conditions was an extreme outlier compared to all other strains, suggesting it may not be representative of the species. Taken together, development of a high-throughput, unbiased approach to quantifying *Candida* biofilm-related phenotypes linked variability in these phenotypes to biofilm production and can facilitate genetic dissection of these critical processes to pathogenesis in the host.

## Introduction

Formation of complex microbial communities, or biofilms, promotes organismal survival by resisting stress and providing a sheltered replicative niche for bacterial, archaeal, and eukaryotic microbes^1^. These structured communities of microbes are embedded within an extracellular matrix that stabilizes the local environment and limits exposure of cells to destructive elements including desiccation, UV radiation, and heavy metals^2–5^. In mammalian hosts, microbial biofilms also provide protection from clearance by host phagocytes and confer resistance to antimicrobial compounds^6–9^. Microbial persistence within human hosts due to biofilm formation is thought to be associated with approximately 80% of human infections caused by bacterial and fungal species^10–14^.

Among fungi, *Candida* species are common members of the human microflora where they reside within the oral, digestive, and genitourinary tracts of the host, as well as on the surface of the skin^15,16^. However, fungal overgrowth of these commensal niches can lead to debilitating superficial infections of the mucosa and skin, as well as systemic or deep-tissue infections that are associated with high mortality rates approaching 50%^17,18^. These infections are particularly common in patients with implanted medical devices, including prosthetics, heart valves, dentures, and catheters, which are susceptible to colonization and biofilm formation by *Candida* species. Patients with catheter-associated biofilms are particularly vulnerable to fungal dissemination and systemic disease because the cells released from the biofilm have immediate access to the host circulatory system^19–21^. Among *Candida* species, *C. albicans* is most commonly isolated from colonized medical devices, although many *Candida* species can form clinically-relevant biofilms on both host biotic surfaces and the abiotic surfaces of implanted devices^22^.

Extensive work has detailed the morphological progression of biofilm formation in *C. albicans*. This highly structured biofilm requires the contribution of both yeast and hyphal cell morphologies^23–25^ and reaches maturity through four major sequential stages that can be observed both in vitro and in vivo^23,26,27^. First, yeast cells adhere to a surface via adhesins (e.g., *EAP1* and the *ALS* gene family) that bind to a broad range of substrates and host ligands^28–31^. Following adherence, yeast cells initiate hyphal production, invading into the substrate and projecting aerially. Extracellular matrix composed primarily of glycoproteins is secreted during establishment of this complex, interwoven hyphal meshwork that encapsulates the entire community^32^. Finally, yeast-like disperser cells are released from aerial hyphae protruding through the extracellular matrix of the mature biofilm that are primed to establish new biofilms at distant locations, leading to disseminated disease^25,28,33^.

Nearly all studies of *C. albicans* biofilm-associated phenotypes have been performed in the genome reference strain, SC5314, with only a few investigations including other clinical isolate backgrounds^34–36^. Recently, key differences in the genetic regulation of biofilm production across *C. albicans* strain backgrounds have suggested that current understandings of biofilm production may be fairly limited^34^. Indeed, analysis of strain collections have revealed significant variation in biofilm and filamentation phenotypes among isolates^36,37^.

Both solid and liquid substrate models have been used to study the biofilm-associated phenotypes of adhesion, filamentation, and invasion in *C. albicans*^33,38–40^. The classical method for simultaneously assaying these phenotypes uses a solid agar substrate to allow filamentation of *C. albicans* colonies, followed by rinsing the agar surface to assess colony adherence, and then physical removal of adherent colonies to reveal invasion profiles into the agar substrate. However, the genetic and environmental regulation of these biofilm-associated traits is often investigated without including their impact on biofilm formation^38,41,42^. Consequently, the relative importance of each process to establishment and maturation of biofilms remains somewhat obscure, although it is clear that mutants defective in adhesion or filamentation produce weak and stunted biofilms^43–45^.

A major hurdle to dissecting biofilm-associated traits has been the lack of quantitative methods available to score each component phenotype. Most mutant studies for adhesion, filamentation, and invasion have relied on visual representations, descriptive language, and other qualitative measures. However, the introduction of the morphology (M) score for agar-based plate assays vastly improved quantitative assessment of colony filamentation^46^. The M score assigns each strain a relative score on a scale from −3 to +3 for center and peripheral colony filamentation, indicating decreased or increased filamentation, respectively, where a reference strain is set to zero. Subsequent adjustments to the M score provided an absolute scale for assigning filamentation but retained numerous caveats (unequal weighting of hyphal production, time-intensive analysis, and use of a colony subset for scoring) and did not include other biofilm-associated phenotypes such as adhesion or invasion^33,37^. Screening adhesion phenotypes using high-throughput methods has also contributed to defining the genes that promote this process but relied on relative quantification within mutant pools, similar to the M score^47,48^.

Here, we describe an automated approach to quantify biofilm-associated phenotypes including adhesion, invasion, and filamentation using the canonical agar-based assay. Application of this method to a collection of 23 characterized *C. albicans* clinical isolates revealed significant variation in adhesion, invasion, and filamentation on solid media. Media but not temperature was the primary determinant of isolate phenotypes across strain backgrounds. Furthermore, the cumulative strengths of adhesion, filamentation, and invasion correlated with biofilm production under some conditions, suggesting a predictive model of biofilm formation exists based on its component phenotypes. Finally, the genome reference strain, SC5314, was a clear outlier among assayed strains across phenotypes, suggesting that current understanding of biofilm-related phenotypes and biofilm formation may yet be limited to the genome reference strain and less applicable across *C. albicans* isolates.

## Results

Previous investigation of filamentation on Spider medium and biofilm formation across a characterized set of 21 *C. albicans* clinical isolates revealed wide phenotypic breadth between strains^37^. Consequently, we centered this study on those 21 isolates and two additional strains, 529L^49,50^ and WO-1^51–53^, which have noteworthy relevance to *C. albicans* pathogenesis and long-standing historical use. Together, these strains are a representative collection of isolates with significant genetic diversity to investigate variation in adhesion, invasion, and filamentation and their relative contributions to biofilm production.

### Development of an automated pipeline for quantitative biofilm-related phenotyping

To assess biofilm-associated phenotypes among *C. albicans* strains, we used the common agar plate-based assay that simultaneously allows measurement of colony filamentation, adhesion, and invasion of the substrate^54^. Each isolate was plated to 100 colonies on three solid agar media: Lee’s, Spider, and YPD, and allowed to grow at either 30 °C or 37 °C for seven days. These media are ordered by increasing nutrient content: Lee’s media contains amino acids as the primary carbon source, Spider provides primarily carbohydrates for growth, and YPD (yeast extract, peptone, dextrose) is a rich complete media.

**Fig. 1:**
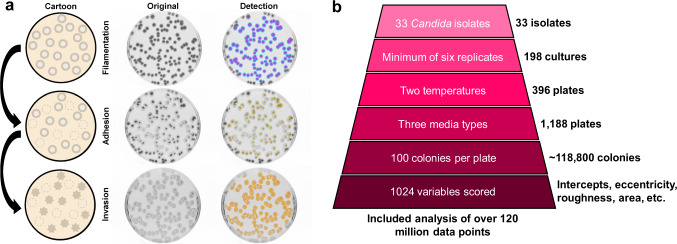
Experimental approach for determining biofilm-related phenotypes. **a** One hundred cells from each strain were plated, allowed to grow for seven days, and the resulting colonies were imaged for filamentation (radial in cyan and center colony wrinkling in magenta). Plates were then rinsed with a stream of water and imaged for adhesion (yellow). Remaining colonies were wiped off and the plates imaged for agar invasion (orange). A cartoon, raw image, and detection overlay (left to right) has been shown to highlight this process. **b** Automated quantification facilitated measurement of ~118,800 colonies to collect over 120,000,000 data points (*C. albicans* – ~85 million, non-albicans *Candida* species – ~35 million) for analysis of filamentation, wrinkling, adhesion, and invasion.

After seven days of growth, plates were imaged from above, and the resultant images were interrogated with custom scripts to detect radial filaments (Fig. 1a, cyan and Supplemental Methods). The degree of radial filamentation was determined as (area_hyphal growth _− area_center colonies_)/(area_center colonies_) to produce an absolute measurement of filamentation that includes all hyphal projections at pixel resolution. Filamentation within the central colony or ‘wrinkling’ was also measured by quantifying colony roughness along a gradient from the smooth, domed appearance characteristic of yeast-dominant colonies to a progressively wrinkled morphology due to the increased prevalence of hyphae (Fig. 1a, magenta). Following acquisition of this first image, the agar plate was washed with a constant stream of water to remove non-adherent colonies, allowed to dry, and then reimaged to measure isolate adherence (Fig. 1a, yellow). Adhesion was calculated as the (area_colonies post-wash_)/(area_colonies pre-wash_), where area is defined by the summed pixel value of every colony on a plate. Finally, we detected colony invasion of the substrate by rubbing the agar surface with a gloved finger under a stream of water to remove any remaining adherent colonies, allowing plates to dry, and acquiring a third image from which invasion was calculated as the (area_hyphal agar invasion_)/(area_colonies pre-wash_) (Fig. 1a, orange). This imaging approach for 23 *C. albicans* strains produced a robust dataset of approximately 85 million data points for this species across these three biofilm-related phenotypes (Fig. 1b).

### *Candida* filamentation is largely influenced by the nutritional environment

**Fig. 2:**
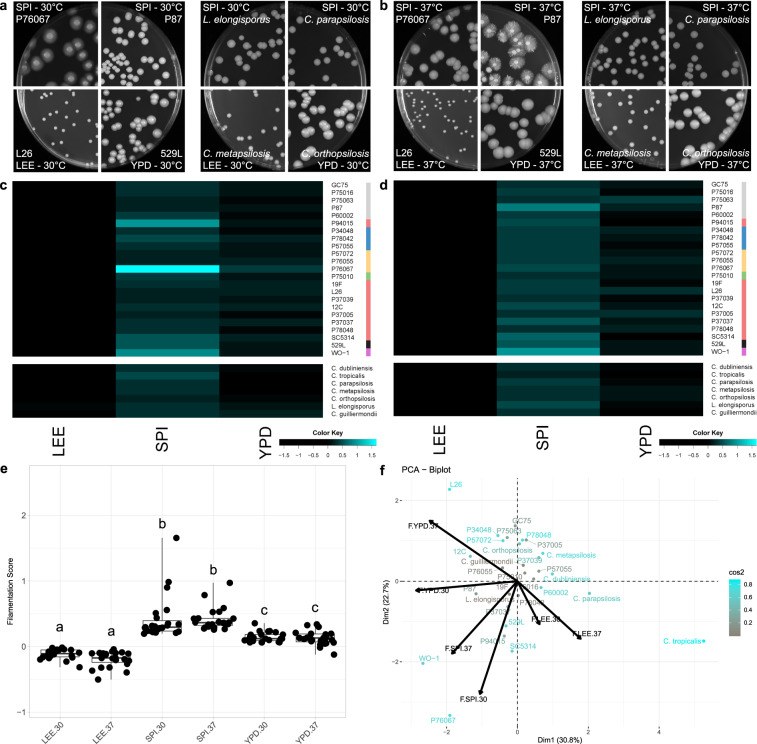
*Candida* radial filamentation is largely defined by medium. Plate images of *C. albicans* isolates (left) and non-*albicans Candida* species (NACS) (right) highlight the varied radial filamentation profiles across media at 30 °C (**a**) and 37 °C (**b**). Heatmaps represent the degree of radial filamentation for *Candida* isolates at 30 °C (**c**) and 37 °C (**d**). Scoring for radial filamentation was defined by (area_hyphal growth _− area_center colonies_)/(area_center colonies_) for at least six biological replicates and averaged. Isolate clade is color coded on the right as in Hirakawa et al.^37^. **e** Radial filamentation scores for each *C. albicans* isolate were grouped and plotted for each media and temperature combination. Overlaid box plots cover the upper and lower interquartile ranges with the intervening line indicating the mean. Whiskers extend to the extreme datapoints. Letters indicate statistically different groups based on Bonferroni-corrected *p* < 0.05 by Dunn’s test. **f** PCA biplots were constructed using radial filamentation data for all assayed *Candida* isolates. Coloration from gray to cyan represents the power of the strain in defining PC1 and PC2. Black arrows represent vectorized phenotypic contributions where the arrows are labeled (Phenotype.Media.Temperature). LEE = Lee’s medium, SPI = Spider medium, YPD = Yeast Peptone Dextrose medium.

*C. albicans* isolates displayed a wide range of filamentation phenotypes across solid media (Fig. 2a, b), ranging from profuse filamentation (P76067-Spider medium at 30 °C) to a complete absence of radial filaments (529L–YPD at 30 °C or 37 °C). Such differential responses between isolates across environments suggests a complex interplay between the assay conditions used and each genetic background. For example, P94015 produced a strong radial filamentation response on solid Spider medium at 30 °C, despite lacking the major filamentous activator *EFG1* and not filamenting on YPD at 37 °C (Fig. 2c, d, S1). Interestingly, media but not temperature strongly influenced filamentation across all *C. albicans* isolates (Fig. 2c–e, Table S1). Specifically, Lee’s medium did not elicit strong radial filamentation whereas multiple strains grown on YPD and Spider media formed abundant hyphae at both 30 °C and 37 °C. Elevated temperature had a variable effect of increasing or decreasing filamentation that was isolate-specific and lacked any clear trends.

To identify isolate characteristics that may associate with filamentation, strains were ordered by their averaged filamentation score for each condition. Rank order plots show most strains filament similarly within each condition (Fig. S2). Surprisingly, the oral isolate 529L, which has been previously described as failing to filament under most conditions^55^, clustered towards the more robust responses on Lee’s and Spider media at 30 °C and 37 °C (Fig. S2). This was not a consequence of its clade or body site of isolation, as neither factor correlated with filamentation among these strains (Fig S3).

Ten non-albicans *Candida* species (NACS) were included in this study to test the utility of this approach beyond *C. albicans* and define their phenotypic responses^53^. In general, NACS displayed weaker filamentation responses than *C. albicans* and only formed radial hyphae when grown on Spider medium (Fig. 2a–d). Similar to *C. albicans*, only media shaped NACS filamentation (Table S1). Importantly, some *Candida* species proved incapable of growing under these conditions. *Debaryomyces hansenii* did not grow at 37 °C, consistent with its primary identity as an environmental fungus^56^, and Lee’s medium was unable to support the growth of *C. glabrata* and *C. lusitaniae*. These species were therefore removed from further investigation.

Previous iterations of the M score to measure *C. albicans* filamentation have included relative quantification of colony center wrinkling^37,44^. Among these 23 *C. albicans* isolates and seven NACS, only SC5314 colonies showed prominent wrinkling under any condition (Fig. S4). Therefore, this morphological response is a unique attribute of the SC5314 background that is either much less frequent or entirely absent in other *Candida* isolates.

### *Candida* filamentation responses are not strongly correlated between solid and liquid substrates

The substrate context of identical media (i.e., solid or liquid) can alter filamentation responses of isogenic strains^57,58^. To determine if filamentation phenotypes are similar in liquid and solid agar media, six *C. albicans* isolates from different clades were grown as overnight liquid cultures in YPD at 30 °C, inoculated into four fresh liquid media (Lee’s, Spider, YPD, and RPMI 1640) at 30 °C or 37 °C, imaged after one and four hours, and scored for the frequency of yeast and hyphae in each image using detection scripts developed for single cell analysis (Fig. S5). Filamentation responses in YPD medium at 30 °C correlated significantly with four of the 16 tested liquid filamentation responses although none were similar solid medium conditions (YPD, 30 °C). In fact, Spider medium at 30 °C was the only condition to elicit similar filamentation responses in liquid and solid substrates (Fig. S6, S7). This suggests that the context of the medium and temperature are important components to activating filamentation responses [59].

### Rich nutrient conditions promote *Candida* species adhesion

**Fig. 3:**
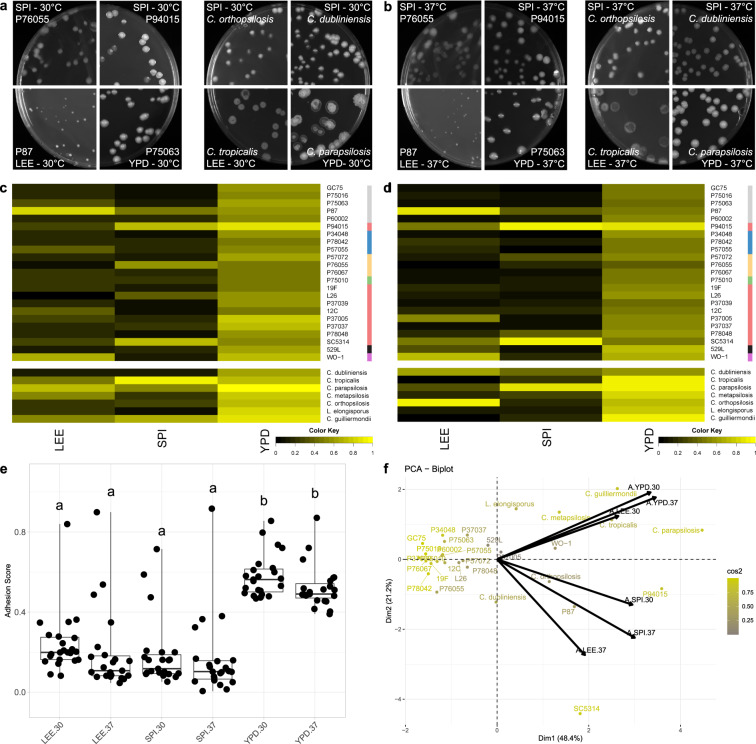
*Candida* adhere to substrates in rich media conditions. Plate images of *C. albicans* isolates (left) and non-*albicans Candida* species (NACS) (right) highlight the varied adhesion profiles across media at 30 °C (**a**) and 37 ^o^C (**b**). Heatmaps represent the degree of agar adhesion for *Candida* isolates assayed for agar adhesion at 30 °C (**c**) and 37 °C (**d**). Scoring for adhesion is defined by the (area_colonies post-wash_)/(area_colonies pre-wash_). Isolate clade is color coded on the right as in Hirakawa et al.^37^. **e** Agar adhesion scores for each *C. albicans* isolate were grouped and plotted for each media and temperature combination. Overlaid box plots cover the upper and lower interquartile ranges with the intervening line indicating the mean. Whiskers extend to extreme datapoints. Letters indicate statistically different groups based on Bonferroni-corrected *p*-values <0.05 using Dunn’s test. **f** PCA biplots were constructed using adhesion data for all assayed *Candida* isolates. Coloration from gray to yellow represents the power of the strain in defining PC1 and PC2. Black arrows represent vectorized phenotypic contributions where the arrows are labeled (Phenotype.Media.Temperature). LEE = Lee’s medium, SPI = Spider medium, YPD = Yeast Peptone Dextrose medium.

Substrate adherence is the critical initial step during biofilm formation to establish a focal point of cellular aggregation and hyphal initiation^25^. To assay isolate adherence, a constant stream of water was applied to colonies on the agar plates used to quantify filamentation, and their ability to remain bound was measured. This set of *C. albicans* isolates displayed highly variable adherence that was largely dependent on media (Fig. 3a, b, S2, Table S1). While most strains remained adhered to YPD agar at both 30 °C and 37 °C (Fig. 3c, d, S8), adherence on Lee’s and Spider media varied widely for *C. albicans* isolates (e.g., P76055 v. P94015 on Spider medium at 37 °C; Fig. 3b, d). In fact, P94015 displayed the strongest adherence of any strain across all assayed conditions (Fig. S8). Adherence was independent of colony size; some isolates, producing small colonies, remained tightly bound to the agar while others, forming large colonies, could be completely washed away (Fig. 3a, b). Surprisingly, adherence decreased at higher temperatures across all three media types (Table S1), but this temperature-dependent regulation was much weaker than the influence of media (Fig. 3e).

Adhesion of NACS was also largely dependent on media. As with *C. albicans*, most species adhered to YPD but not Spider or Lee’s media, but no association was found with temperature (Fig. 3a–d). In a number of cases, NACS adhesion was stronger than that observed for *C. albicans* isolates (e.g., YPD at 37 °C). Principal component analysis (PCA) of adhesion highlighted these differences between *C. albicans* strains and NACS. Most *C. albicans* isolates clustered together, whereas NACS occupied a large segment of the two-component space (Fig. 3f), highlighting their relatively wide range of adherence phenotypes.

### *C. albicans* and NACS differ in agar invasion

**Fig. 4:**
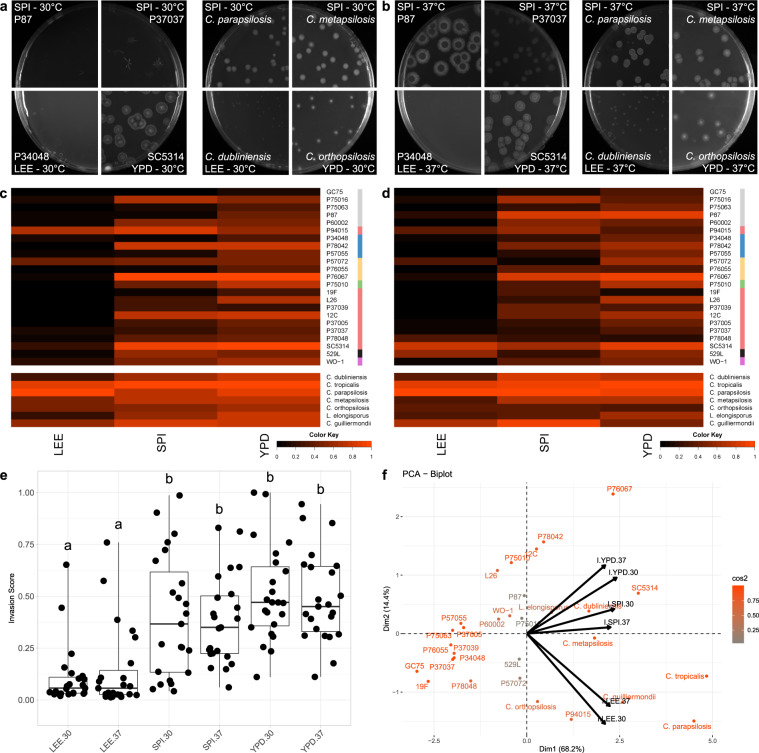
*Candida* isolates display high variability in substrate invasion. Plate images of *C. albicans* isolates (left) and non-*albicans Candida* species (NACS) (right) show resultant invasion profiles across media at 30 °C (**a**) and 37 °C (**b**). Heatmaps represent the degree of agar invasion for *Candida* isolates assayed for agar invasion at 30 °C (**c**) and 37 °C (**d**). Agar invasion was calculated as the (area_hyphal agar invasion_)/(area_colonies pre-wash_). Isolate clade is color coded on the right as in Hirakawa et al.^37^. **e** Agar invasion scores for each *C. albicans* isolate were grouped and plotted for each media and temperature combination. Overlaid box plots cover the upper and lower interquartile ranges with the intervening line indicating the mean. Whiskers extend to extreme datapoints. Letters indicate statistically different groups based on Bonferroni-corrected *p*-values <0.05 using Dunn’s test. **f** PCA biplots were constructed using radial filamentation data for all assayed *Candida isolates*. Coloration from gray to orange represents the power of the strain in defining PC1 and PC2. Black arrows represent vectorized phenotypic contributions where the arrows are labeled (Phenotype.Media.Temperature). LEE = Lee’s medium, SPI = Spider medium, YPD = Yeast Peptone Dextrose medium.

To assess agar invasion, all remaining adherent colonies were removed with a gloved finger under running water, and the imprints into the agar from colony invasion were imaged. Conditions that did not produce robust filamentation (Lee’s medium) also failed to induce invasion with the exception of P94015 and SC5314, which strongly invaded the agar substrate on Lee’s medium at 30 °C despite neither having produced radial hyphae (Fig. 4a–d, S9). In fact, P94015, the isolate lacking the filamentation activator *EFG1*, invaded the agar proficiently across all tested conditions (Fig. 4c, d, S2). As with the other biofilm-related phenotypes, isolates produced a range of invasion profiles from a complete lack of agar invasion to strongly invasive across media and temperature (e.g., P87 and 529L; Fig. 4a–d). Similar to filamentation, media significantly altered colony invasion (Fig. 4e, Table S1), but temperature did not. Yet, in contrast to the other phenotypes, *C. albicans* isolates exhibited a continuum of invasion phenotypes across most media instead of a cluster of phenotypically similar strains with a handful of outliers (Fig. S3).

Unlike most *C. albicans* isolates, NACS invaded the agar media proficiently across all tested conditions, including Lee’s media at 30 °C and 37 °C (Fig. 4a–d). This difference between *C. albicans* and NACS is particularly evident on PC1 of an ordination plot that separates most *C. albicans* isolates from the NACS, with the exception of the invasive strains P94015 and SC5314 (Fig. 4f).

### *Candida albicans* biofilm formation is a multifactorial process

To compare biofilm-related phenotypes of *C. albicans* isolates to total biofilm production under the same media and temperature conditions, we seeded cells into 96-well plates and allowed biofilms to form in Lee’s, Spider, and YPD media at 30 °C and 37 °C for 24 h^59^. RPMI 1640 medium supplemented with 10% FBS was also included as a common condition to assay biofilm formation. Biofilm production was measured as the optical density (OD_600_) of each well after rinsing to remove non-adherent cells.

**Fig. 5:**
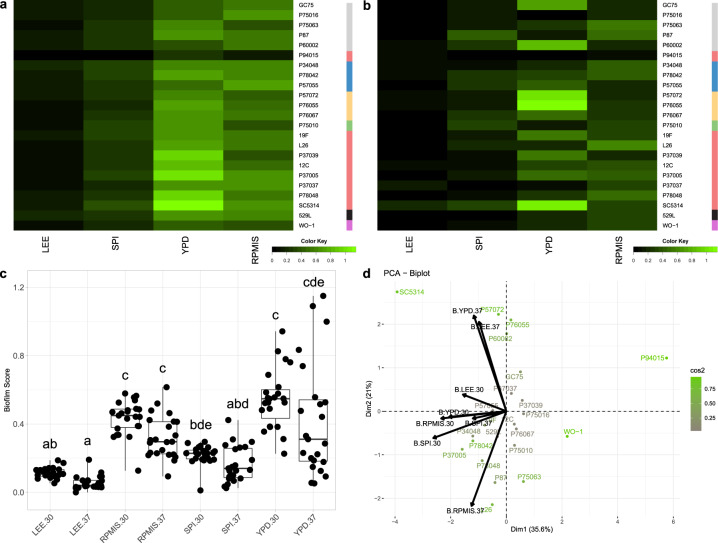
Biofilm formation increases with media richness across *C. albicans* isolates. Biofilms were seeded in a 96-well high-throughput assay and measured by OD_600_ after 24 h. Heatmaps represent the total biofilm biomass for *C. albicans* isolates at 30 °C (**a**) and 37 °C (**b**). Isolate clade is color coded on the right as in Hirakawa et al.^37^. **c** Biofilm formation scores for each *C. albicans* isolate were grouped and plotted for each media and temperature combination. Overlaid box plots cover the upper and lower interquartile ranges with the intervening line indicating the mean. Whiskers extend to the extreme data points. Letters indicate statistically different groups (Bonferroni-corrected *p*-values <0.05) using Dunn’s test. **d** PCA biplots were constructed using radial filamentation data for all assayed *Candida* isolates. Coloration from gray to green represents the power of the strain in defining PC1 and PC2. Black arrows represent vectorized phenotypic contributions where the arrows are labeled (Phenotype.Media.Temperature). LEE = Lee’s medium, SPI = Spider medium, YPD = Yeast Peptone Dextrose medium. RPMIS = RPMI 1640 supplemented with 10% FBS.

The amount of biofilm produced by *C. albicans* was strongly dependent on media and temperature (Fig. 5c). Biofilm mass increased in richer media and was generally greater at 30 °C than at 37 °C (Table S1). Thus, increasing the media temperature to 37 °C led to little biofilm production for almost half of the *C. albicans* strains in any medium and more stochastic biofilm production among the remaining strains in YPD, which induced the largest biofilms at 30 °C (Fig. 5a, b, S10). RPMI 1640 + 10% FBS (RPMIS) produced a phenotype intermediate to Lee’s/Spider media and YPD at both tested temperatures. As with other biofilm-related traits, P94015 and SC5314 displayed aberrant phenotypes (Fig. 5d); P94015 failed to form a biofilm in any condition and SC5314 produced among the most robust biofilms in all tested conditions.

**Fig. 6:**
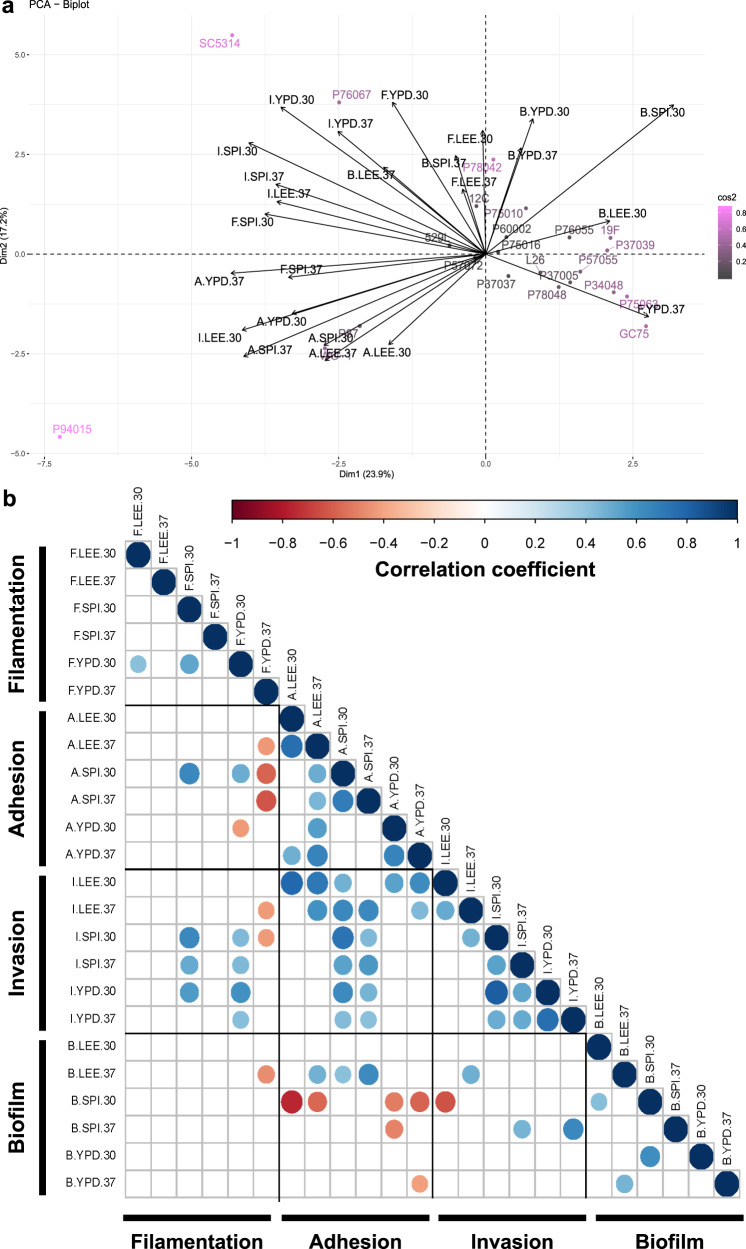
Most biofilm-associated phenotypes do not correlate. **a** A PCA biplot was constructed using the aggregate data for *C. albicans* filamentation, adhesion, invasion, and biofilm production. Coloration of isolate names from gray to orchid indicate the relative contribution of each isolate to determining the coordinate axis. Black arrows indicated vectorized phenotypic contributions where the arrows are labeled (Phenotype.Media.Temperature). **b** Each biofilm-related phenotype was tested for correlations to all other phenotypes by Pearson’s correlation. Blue indicates a significant positive correlation and red indicates a significant negative correlation at an adjusted *p*-value < 0.05. Phenotypes are labeled as (Phenotype.Media.Temperature).

The aggregated data for all strains was plotted as principal components and overlaid with phenotypic vectors for filamentation, adhesion, invasion, and biofilm production (Fig. 6a). Some strains are clearly defined by specific biofilm-related phenotypes that distinguish them from others within this set. For example, P87 resides within the quadrant largely occupied with vectors for adhesion, and P76067 segregates from other strains due to its strong radial filamentation and condition-specific invasion patterns. Importantly, P94015 and SC5314 are clear outliers for biofilm production and its component processes, likely reflecting the combination of aberrant biofilm-related phenotypes compared to most *C. albicans* strains. Invasion vectors tend to reside midway between adhesion and filamentation (Fig. 6a), consistent with the degree of correlations found within these phenotypes (Fig. 6b). Biofilm vectors generally cluster away from the other phenotypes and show no correlation to most biofilm-associated phenotypes (Fig. 6b). Taken together, this indicates that biofilm formation cannot be predicted by any single biofilm-associated phenotype.

**Fig. 7:**
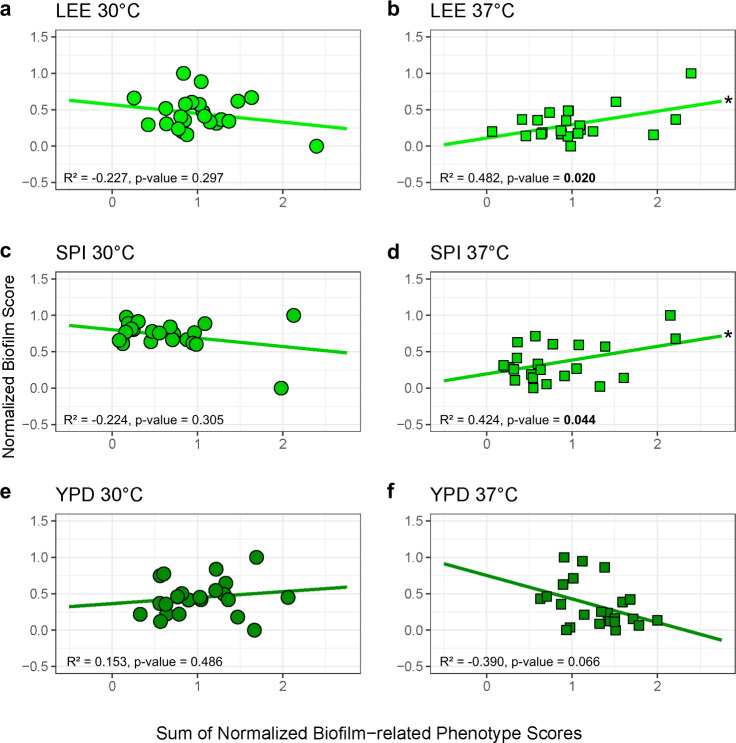
Cumulative biofilm-related phenotype scores correlate with biofilm production. Normalized filamentation, adhesion, and invasion scores were summed for each tested strain and plotted (*y*-axis) against the biofilm score (*x*-axis) for isolates at 30 °C (LEE (**a**), SPI (**c**), YPD (**e**)) and 37 °C (LEE (**b**), SPI (**d**), YPD (**f**)).

To test the integrated contributions of multiple biofilm-related phenotypes to total biofilm production, the relative scores for each component phenotype were weighted equally and summed to generate a composite score that was plotted against biofilm measurements for each media and temperature condition (Fig. 7, Table S2). The composite score for biofilm-related phenotypes and biofilm production in Lee’s and Spider media at 37 °C significantly correlated (Lee’s: Pearson’s test = 0.48, df = 22, *p*-value = 0.02, Spider: Pearson’s test = 0.42, df = 22, *p* = value = 0.04). Weighting individual biofilm-related phenotypes slightly improved but did not substantially change these associations (Fig. S11, Table S3). Thus, the additive contributions of adhesion, filamentation, and invasion are predictive of the degree of biofilm production under some conditions.

## Discussion

Development and implementation of a highly quantitative approach to investigate biofilm-associated processes revealed significant variation among *C. albicans* clinical isolates and non-albicans *Candida* species. These responses were largely dependent on the nutrient conditions of the agar substrate. In addition, this variability facilitated construction of an additive model of total biofilm production, where the equivalent contributions of an isolate’s biofilm-related phenotypes correlated strongly with biofilm biomass in Lee’s and Spider media at 37 °C. Finally, two strains, P94015 and the genome reference strain, SC5314, displayed aberrant phenotypes compared to most *C. albicans* isolates, distinguishing them as outliers in biofilm-associated responses.

The automated approaches developed and implemented here greatly enhance investigation of *C. albicans* biofilm-related phenotypes. Previous work on *Candida* adhesion and invasion has been mostly descriptive, with the notable exception of adhesion in microtiter plates for large-scale mutant selection and the M score^46,47^. Even with the iterative developments of the M score to increase the reproducibility, ease, and precision of quantifying filamentation^37,44,54^, the labor and time necessary to perform genome-wide mutant screens or large strain collections for these phenotypes have remained a strong deterrent. The analysis pipelines we developed can greatly facilitate these large-scale efforts and remove experimental bias by including all colonies in analysis of 1000s of plate images per hour, shifting the burden of experimentation from quantifying strain phenotypes to the experimental setup required to phenotype strains of interest.

This approach was designed with the goal of being suitable for analysis of all *Candida* species as NACS have been comparatively understudied for these biofilm-associated phenotypes. All NACS in the *Candida* paraphyletic group showed weak radial filamentation, robust agar invasion, and mostly consistent adherent properties that were dependent on media type. Yet, biofilms produced by NACS have vastly different structures that, in some cases, do not include hyphal cells^20^. This points to either additional or alternative factors that regulate biofilm regulation within these species and/or the different contributions of biofilm-related phenotypes to the lifestyle and success across *Candida* species.

Phenotypic variability among isolates is a characteristic feature of *C. albicans*^37,60,61^ and is further supported by our investigation of biofilm-related phenotypes. Some of this variability is likely due to inactivating mutations in key regulators of filamentation and biofilm formation (e.g., *EFG1* in P94015^37^). However, the genetic basis for the majority of this variation remains unknown, suggesting that additional, potentially unknown loci contribute to phenotypic variation among these strains, as was recently reported for biofilm formation^34^. Preliminary analysis of gene expression across 21 *C. albicans* represented in this study found differential expression of genes encoding components of the cell wall and cell membrane when grown under identical conditions that may contribute to these phenotypic differences (manuscript under review). Of particular interest will be the strong adhesion phenotype of P94015, which lacks the hyphal-regulator *EFG1*, and condition-dependent filamentation by *C. albicans* isolate 529L, which had been previously described as being incapable of forming true hyphae^55^.

The nutrient environment in which strains are grown strongly impacts biofilm-related phenotypes. Spider medium, which is not a rich media and provides mannitol as the primary carbon source, is commonly used to induce filamentation^37,62^. Indeed, multiple strains display prominent radial filamentation on this medium, but this response is not simply due to the reduced availability of carbon, as growth on amino acid-rich Lee’s medium induced little to no hyphal production across *Candida* species. Yet, in a previous study, Lee’s medium proved to be the most common medium to detect filamentation defects when screening a genetic deletion library across ten in vivo filamentation conditions^58^. It is possible that genetic variation in the SC5314 genetic backgrounds or differences in media components could underlie these discrepancies^37,63^. In addition, our data suggests that media content dictates filamentation responses more than growth in liquid or on a solid substrate, even when those contacts activate different genetic pathways^58^. This variation in responses to medium type suggests that use of multiple media in characterizing mutant strains may aid in determining the universal or conditional role of that gene in filamentation and other biofilm-related phenotypes. In addition, recent work highlighting the role of mechanosensing and agar density on microbial invasion and adherence suggests substrate composition may also play a role in *Candida* biofilm formation^64–66^.

This work also expands beyond filamentation to describe the environmental responses of agar invasion and adhesion. Filamentation and invasion profiles of strains often displayed similar trends, especially between YPD and Spider media (Fig. 6). This may point to similar regulation of hyphal production during the process of biofilm anchoring and generation of aerial hyphae, although these mechanisms likely diverge during the transition to hypoxia as hyphae penetrate further into the substrate^67,68^. Unexpectedly, strains with robust radial projections of hyphae often did not have strong adherence (e.g., P76067). This would suggest that anchoring the biofilm relies on additional activities beyond production of hyphae and their associated repertoire of adhesion molecules^25^. While previous work has noted the dependence of adhesion on media types^39^, trends between rich media and increased adhesion have not been described to the best of our knowledge as was observed here. Finally, this work reveals the surprising result that temperature does not significantly alter the phenotypic outcome of filamentation and invasion using solid agar-based assays. While it is clear that elevated temperature (37 °C) rapidly induces filamentation^69,70^, these assays are commonly conducted in liquid culture with SC5314. Indeed, previous plate-base assays of filamentation showed no clear pattern between hyphal production and temperature^37^. Furthermore, despite a temperature-dependent increase in invasive growth observed for SC5314 in this work, inclusion of clinical isolates that behave differently than the reference strain negated these associations.

Cumulative measures of biofilm-related phenotypes correlated with biofilm production. While these three component phenotypes are known to play important roles in the sequential progression of biofilm maturation, they have not been previously shown to provide predictive power for biofilm potential of individual strains. The lack of correlation under some conditions may represent slight differences in sensing solid substrates used between assays (agar v. polystyrene) or additional environmental factors that are not captured in these assays. This is represented in a lack of significant positive correlations between biofilm-related phenotypes and biofilm formation across half of the assayed condition. Yet, composite phenotype scores on Lee’s and Spider media at 37 °C displayed significant positive correlations with production of biofilm. These findings support the importance of each component to total biofilm production and demonstrate that no one phenotype takes clear precedence over another.

As has been observed in other fungal systems^37,71^, the *C. albicans* reference strain displayed multiple phenotypes that distinguished it from other isolates of the same species. For example, center colony wrinkling, previously assessed as a unique variable of plate based filamentation^33,37,46^, is almost exclusive to the genome reference strain SC5314. In general, SC5314 displayed more prominent phenotypes in nearly all conditions, which may reflect lab adaptation or divergent regulation of these biofilm-associated processes prior to isolation. Regardless, we hypothesize that the genetic circuits controlling these processes in SC5314 have diverged from those in other clinical isolates or are expressed at altered levels to produce more robust phenotypes^34^. If SC5314 pathways have been rewired, focused studies only in the genome reference strain may preclude identification of important phenotypic regulators relevant to clinical infections. Indeed, alterations to the organization of biofilm-associated networks recently described within this strain set^34,72^ may indicate this is a common feature of *C. albicans* isolates. Thus, this work further suggests that multiple strain backgrounds should be included in molecular and phenotypic studies of *C. albicans* to ensure similar phenotypes are produced in mutants or under identical conditions.

Among the most vexing questions from this study surround the ability for all of these isolates to cause clinical disease while displaying extensive phenotypic variation of these pathogenic processes. As the majority of these isolates were obtained from systemic infections, how do strains incapable of filamenting strongly under a range of environmental conditions cross epithelial barriers and disseminate across the host? Difficulty recapitulating the precise conditions within the human host through in vitro assays may be one explanation. Alternatively, it may reflect procedures of clinical isolation where a single colony is usually chosen to represent the entire infective population. Thus, the single colony isolate may not accurately embody the clinical population. It is also possible that the site of isolation is not necessarily the site of initial colonization and persistence that would be under stringent selection prior to population expansion. Finally, robust biofilm-associated phenotypes may not be necessary to produce disease in certain patient populations where the lack of innate immune effectors or other competing sources of inflammation allow *C. albicans* to expand and spread through the host. Investigations focused on the phenotypic diversity found among these *C. albicans* isolates coupled with high-throughput phenotyping will facilitate exploration of the genetic basis for these divergent responses and how massive phenotypic variation can occur for isolates producing similar disease states.

## Methods

### Strains and media used

Strains are listed in Table S4. Altogether, 23 *C. albicans* clinical isolates^37,49–53^ and ten NACS^53^ were included in this study. Four media were used for *C. albicans* growth and phenotyping; LEE (Lee’s medium)^73^, SPI (Spider medium)^74^, YPD (yeast extract, peptone, dextrose), liquid RPMI 1640 without additives (Corning, Corning, NY), and RPMI 1640 supplemented with 10% fetal bovine serum.

### Filamentation, adhesion, and invasion assays on solid media

Overnight cultures for each strain were struck out from −80 °C freezer stocks on YPD solid agar. Individual colonies were picked and transferred into 3 mL of liquid YPD for overnight growth. Overnight cultures were counted using a hemocytometer and diluted into sterile 1× PBS. Diluted cultures were plated to 100 colonies on LEE, SPI, and YPD solid agar plates. Plates were grown for seven days at 30 °C or 37 °C and imaged using a ChemiDoc XRS + imager (Bio-Rad, Hercules, CA) from the top of the plate.

Plate images were scored for radial filamentation and center colony wrinkling using custom scripts described briefly in the detection section below and in detail in the Supplemental Methods. After imaging, each plate was subjected to a flow of water at a 45° angle for ~3 s to remove non adherent cells. Flow rate for the adhesion wash was measured at 31.4 ± 3.6 mL/s dispensed from a 1 cm diameter silicon tube. Plates were allowed to dry and imaged again to score adhesion. Colonies were then directly removed by wiping with a gloved finger under a stream of water and imaged to score for invasion. Adhesion and invasion were also scored using custom visual analysis scripts described below. Each solid plate experiment was conducted in a minimum of six replicates and at least triplicate by two separate researchers.

### Detection of radial filamentation, agar adhesion, and agar invasion

The following description of quantification is given in greater detail in the Supplemental Methods. Images taken through plate-based assays were processed by the visual analysis tool MIPAR, version 3.0.3 (MIPAR, Worthington, OH). The MIPAR recipes [LEE_radial.rcp (Supplemental Data set 1), SPI_radial.rcp (Supplemental Data set 2), and YPD_radial.rcp (Supplemental Data set 3)] were used to detect filamentation on Lee’s, Spider, and YPD media plates. These scripts analyze the image and output a radial filamentation score. Briefly, the plate area was detected, and the edges of the plate were removed to eliminate colonies which contact the edge of the plate. Colonies were then selected within the remaining plate area through a process of smart clustering based on brightness. This clustering step serves the purpose of delineating brightness of the colonies away from the plate coloration. Final radial filamentation was measured as (area_hyphal growth _− area_center colonies_)/(area_center colonies_).

Detection for adhesion was conducted similarly to radial filamentation using the MIPAR script [Adhesion_detection.rcp (Supplemental Data set 4)]. Adhesion detection was applied to both the original image (prior to wash) and the washed image. Adhesion was scored as (area_colonies post-wash_)/(area_colonies pre-wash_). Quantification of agar invasion followed with the MIPAR recipe [Invasion_detection.rcp (Supplemental Data set 5)] to identify the remaining hyphal profile from the darker plate background. Invasion detection was applied to both the original image (prior to treatments) and the image of the plate following direct wiping of all colony material from the plate and scored as (area_colonies agar invasion_)/(area_colonies pre-wash_).

### Liquid filamentation and detection

Overnight cultures of the six *C. albicans* isolates used for liquid filamentation were grown at 30 °C in 5 mL YPD liquid medium. Cultures were separated into 1 mL aliquots, centrifuged at 5000×*g* for 5 min, and washed twice with sterile 1× PBS. Each sample of washed cells was resuspended in 1 mL of target liquid media (Lee’s media, Spider media, YPD, or RPMI), and 100 μL of the resuspension was added to 3 mL of the same media (1:30 dilution). The diluted cultures were then incubated at 30 °C or 37 °C, shaking at 225 rpm for 1 or 4 h. Glass slides were prepared with 10 μL of each culture and visualized across a minimum of eight random fields of view containing at least eight cells using a Leica DM 750 with an attached Leica MC170HD digital camera (Leica, Wetzlar, Germany). Four biological replicates were performed for each strain in each condition.

Images were processed by the visual analysis tool MIPAR, version 3.0.3 (MIPAR, Worthington, OH). The liquid detection recipe [Liquid_detection.rcp (Supplemental Data set 6)] was used to select for darker cells against a lighter background. The total number of cells was then tabulated and binned as true hyphae or non-hyphae, where roundness <0.43 was scored as hyphae. MIPAR produced the counts of yeast, hyphae, and total cells for all images within this set. The recipe files designed for these assays are available for download in the supplement, as well as accessible here.

### Biofilm assay

A high-throughput 96-well format biofilm assay was adapted from^59^. Cultures were grown for 17 h in liquid YPD at 30 °C on a drum roller. Three microliter of overnight culture was added to each well of an untreated F-bottom Cellstar® 96-well plate (Greiner, Monroe, NC) that were preloaded with 197 μL of the desired growth media (LEE, SPI, YPD, or RPMIS) at the desired temperature for a starting OD_600_ of ~0.4. Plates were sealed using a Breathe-Easy sealing membrane (Sigma-Aldrich, St. Louis, MO) and shaken at 250 rpm at 30 °C or 37 °C for 90 min to allow for cell adherence to the bottom of the well. Media was aspirated off, and each well was rinsed with 200 μL of sterile 1× PBS to remove planktonic and non-adherent cells. Two hundred microliter of desired growth media was then added to each well. Plates were loaded into a Synergy H1 microplate reader (BioTek, Winooski, VT) and grown for 24 h, shaking at the double orbital fast setting at 30 °C or 37 °C.

Following biofilm growth, media was aspirated from all wells to remove planktonic cells. Wells in which the biofilm was dislodged were excluded from analysis. OD_600_ was then scanned in a 5 × 5 grid for each well with well edges removed from analysis. Optical density reads for each well were then averaged across two technical replicates to calculate total biofilm formed for each biological replicate. The OD_600_ from wells lacking cells was then subtracted from this average to calculate the average and standard deviation for each strain under each condition. Four biological replicates were used for each strain in each biofilm-growing condition.

### Data analysis and visualization

Analyses were performed in R (version 3.3.2). Heatmaps were generated using the R-package ‘gplots’ with color bars divided into 20 independent segments for visualization. Supplemental figure bar charts were generated using the R-package ‘ggplot2’. PCA visualization and analysis was conducted using the R-package ‘factoextra’ where biplots were generated to show the relative contribution of each phenotype to the primary PCs. Detailed protocols for image detection, scoring, and weighting have been included in the Supplemental Methods.

### Reporting summary

Further information on research design is available in the Nature Research Reporting Summary linked to this article.

## Supplementary information

Supplementary Information

Supplementary Data 1

Supplementary Data 2

Supplementary Data 3

Supplementary Data 4

Supplementary Data 5

Supplementary Data 6

Reporting Summary

## Data Availability

The datasets generated during and/or analyzed during this study are available from the corresponding author on reasonable request. Summary of these datasets are included in the published article.

## Code availability

Code used for data organization and analysis is available through OSU git repositories: https://code.osu.edu/fillinger.22/MIPAR_FWAIBL_Sorted_Score_Files, https://code.osu.edu/fillinger.22/MIPAR_FWAIBL_Normalized_Figures, https://code.osu.edu/fillinger.22/MIPAR_FWAIBL_R2R_Additive_Correlations, and https://code.osu.edu/fillinger.22/mipar_fwaibl-r2r-weighted_phenotypes.
