# Supplementary material for: Automated quantification of Candida albicans biofilm-related phenotypes reveals additive contributions to biofilm production
Source: NPJ Biofilms Microbiomes. 2020 Oct 9;6:36. doi: 10.1038/s41522-020-00149-5 (PMC7547077; doi:10.1038/s41522-020-00149-5)
Supplement: Supplementary file 1 — Supplementary Information [file 41522_2020_149_MOESM1_ESM.pdf]

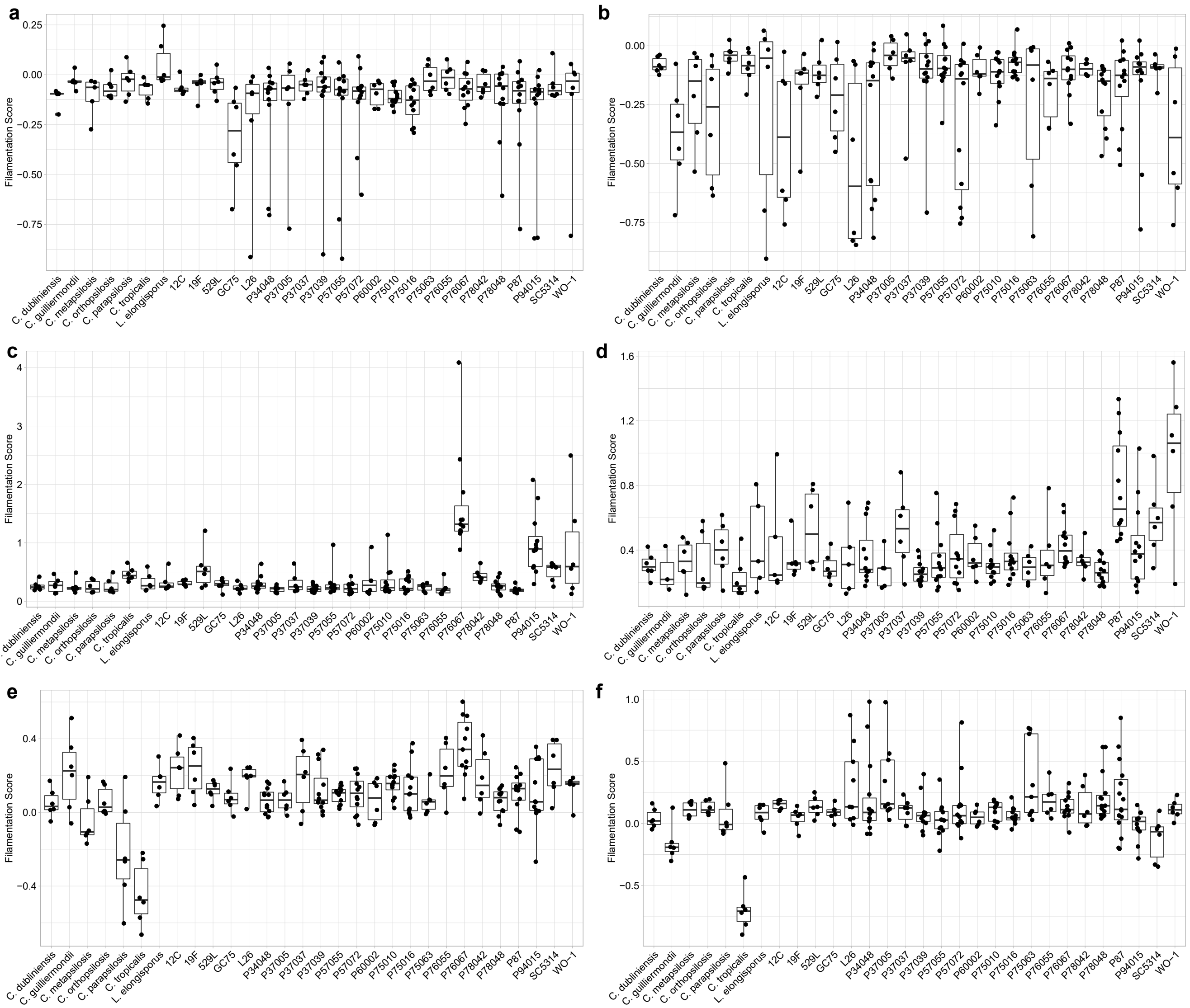

Supplemental Figure 1. Radial filamentation of *Candida* isolates. Radial filamentation data has been plotted for 30°C (LEE (**a**), SPI (**c**), YPD (**e**)) and 37°C (LEE (**b**), SPI (**d**), YPD (**f**)). Overlaid box plots cover the upper and lower interquartile ranges with the intervening line indicating the mean. Whiskers extend +/- two standard deviations. A minimum of six biological replicates were conducted per strain. LEE = Lee's medium, SPI = Spider medium, YPD = Yeast Peptone Dextrose medium.

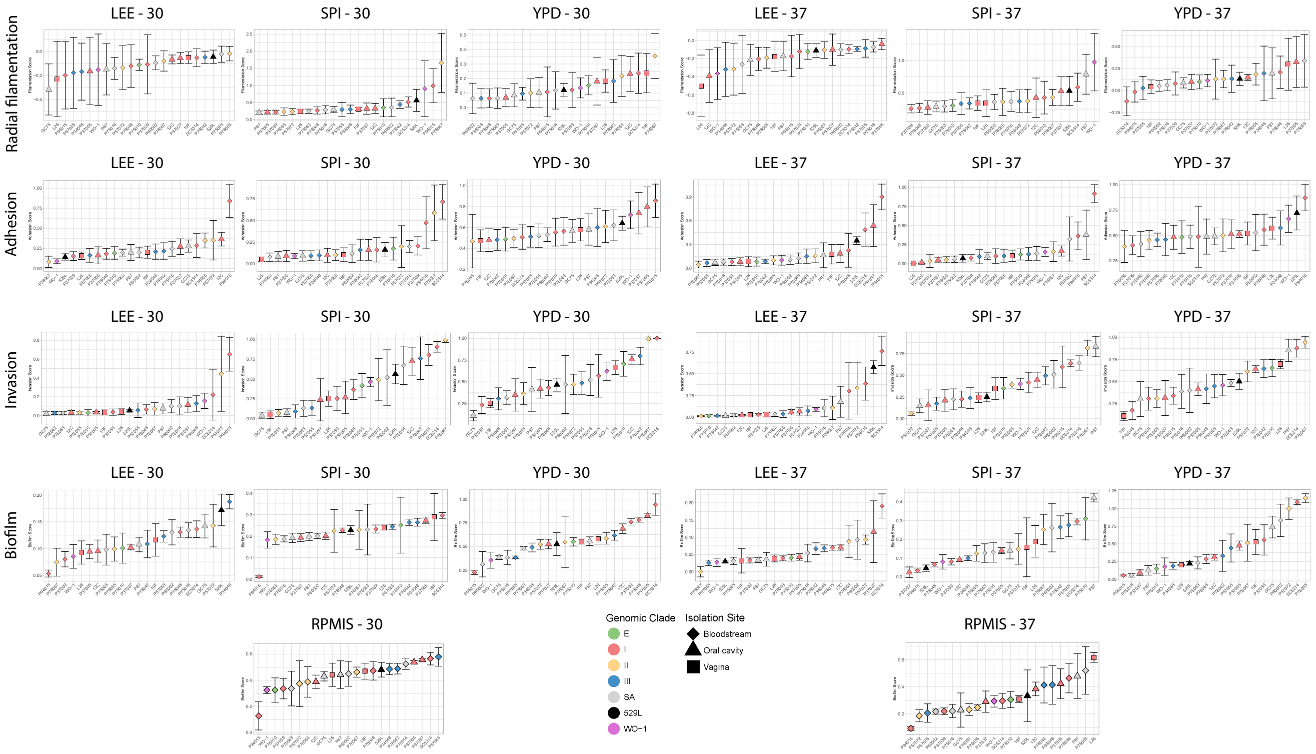

Supplemental Figure 2. Rank order plots for *C. albicans* biofilm-related phenotypes. *C. albicans* strains were ordered by increasing score across all tested conditions. Averaged phenotypic scores for each *Candida* isolate were plotted by fingerprinting clade (coloration) and body site of isolation (symbol) in order by mean. Whiskers extend +/- two standard deviations. LEE = Lee's medium, SPI = Spider medium, YPD = Yeast Peptone Dextrose medium. RPMIS = RPMI 1640 supplemented with 10% FBS.

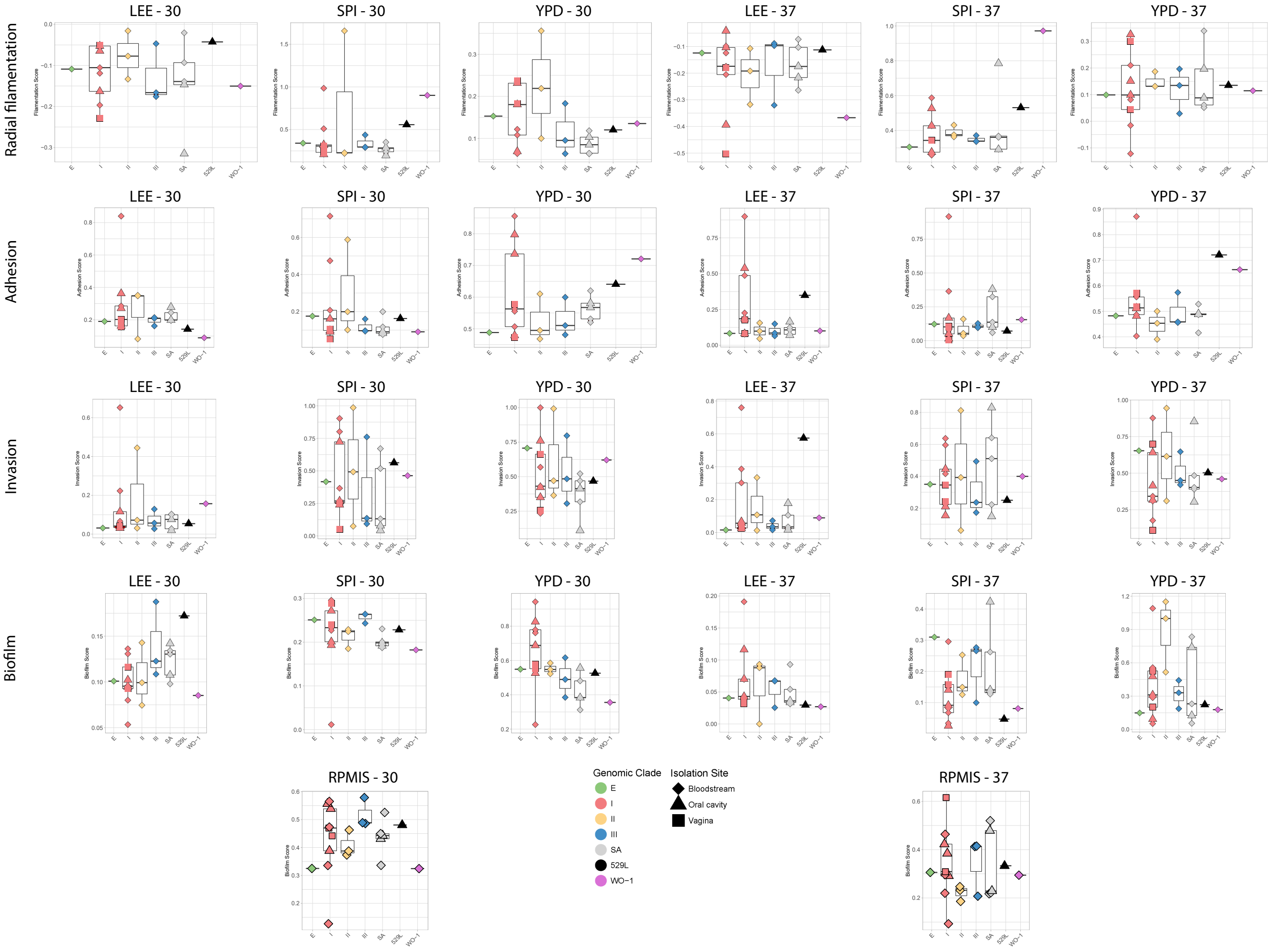

Supplemental Figure 3. Fingerprinting clade and body site of isolation do not associate with biofilm-related phenotypes. Averaged phenotypic scores for each *Candida* isolate were plotted by fingerprinting clade (coloration) and body site of isolation (symbol) for all assayed conditions. Data overlaid box plots cover the upper and lower interquartile ranges with the intervening line indicating the mean. Whiskers extend +/- two standard deviations. LEE = Lee's medium, SPI = Spider medium, YPD = Yeast Peptone Dextrose medium. RPMIS = RPMI 1640 supplemented with 10% FBS.

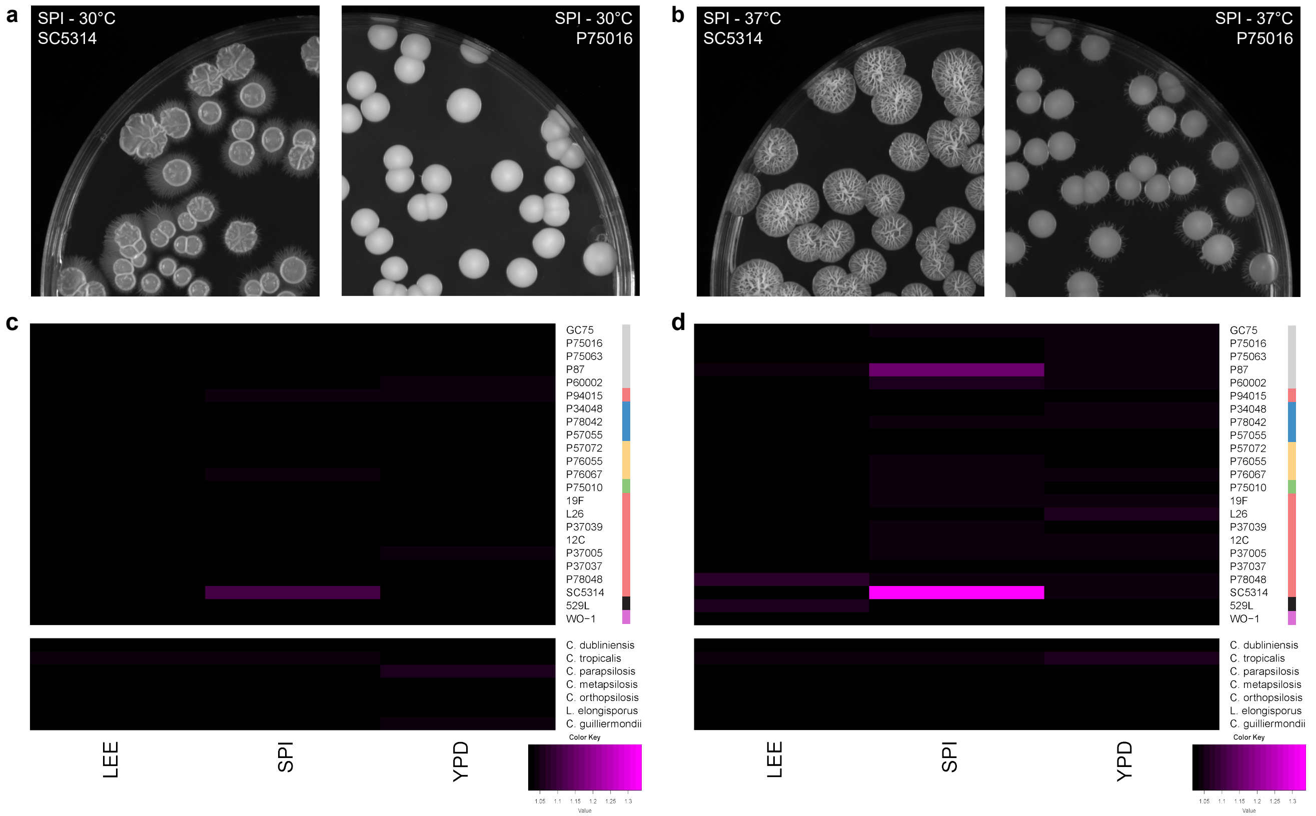

Supplemental Figure 4. *Candida* species colony wrinkling is primarily observed in SC5314. Plate images of *C. albicans* isolate SC5314 (left) and P75016 (right) highlight the varied colony wrinkling profiles across media at 30°C (**a**) and 37°C (**b**). Heatmaps of all *Candida* isolates assayed for colony wrinkling at 30°C (**c**) and 37°C (**d**). Scoring for center colony wrinkling is defined by mean roughness (ratio of area of tightest-fitting convex hull and area of each feature) of the center colonies. Isolate clade is color coded on the right as in Hirakawa *et al*[37]. LEE = Lee's medium, SPI = Spider medium, YPD = Yeast Peptone Dextrose medium.

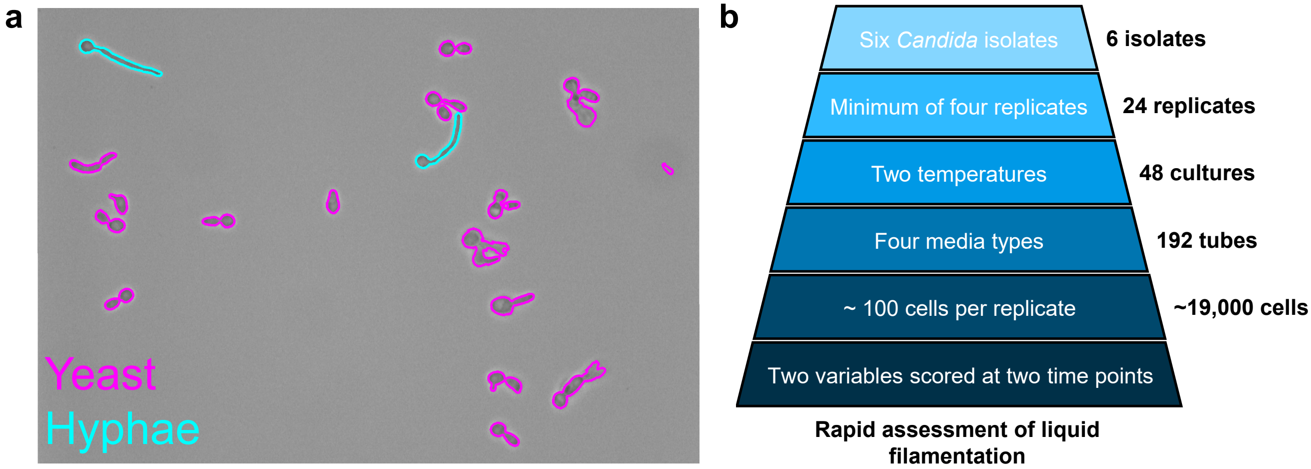

Supplemental Figure 5. Automated detection of *C. albicans* cell morphology. (**a**) MIPAR recipes were developed to detect yeast (magenta) and hyphae (cyan) from microscopy images of cells from liquid culture. (**b**) Automation of cell morphology detection facilitated the scope of this work investigating filamentation in liquid media. Detection results in classification of cells as yeast or hyphae and calculates total pixel area for each condition.

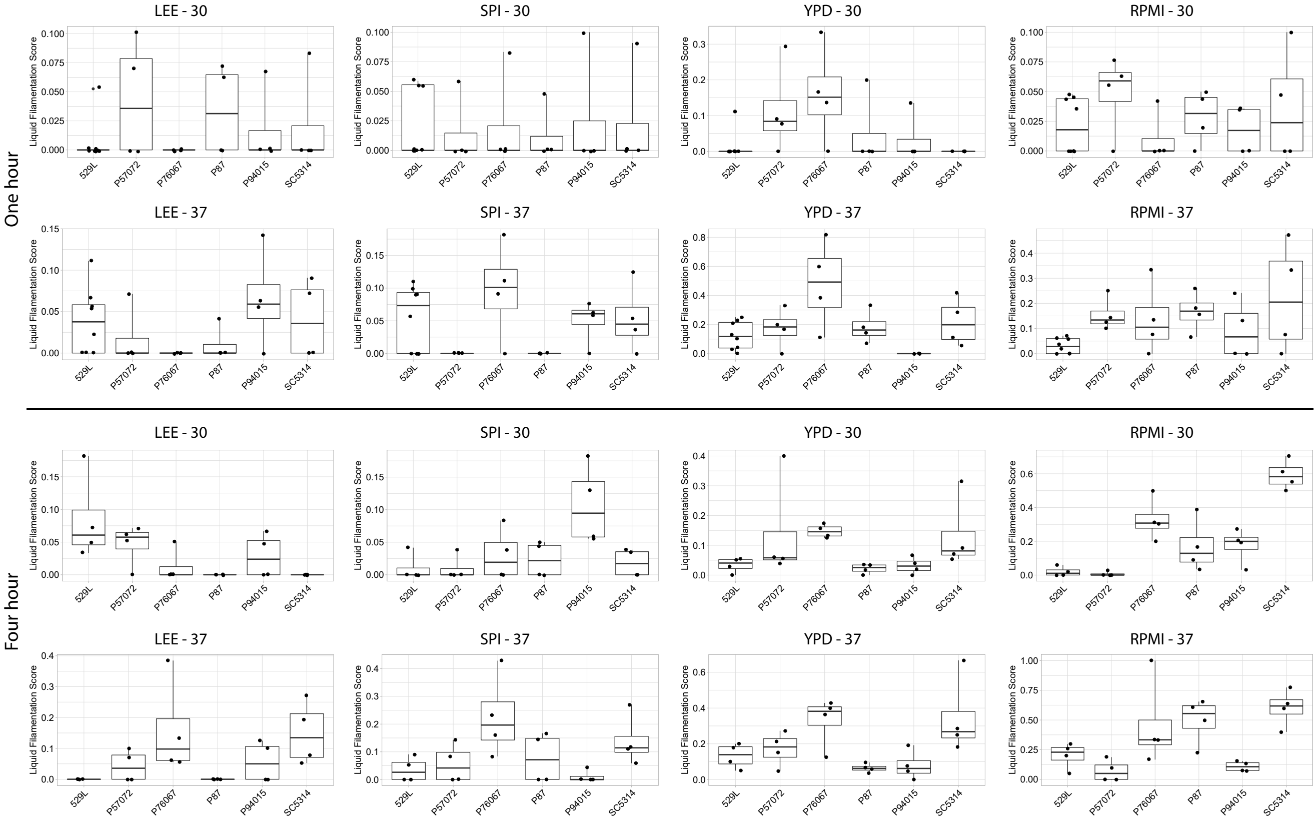

Supplemental Figure 6. *C. albicans* liquid filamentation. *C. albicans* cells were inoculated into liquid media and allowed to grow for one and four hours. The fraction of hyphal cells was determined across a minimum of eight fields of image per replicate and plotted for 30°C and 37°C. Overlaid box plots cover the upper and lower interquartile ranges with the intervening line indicating the mean. Whiskers extend to the most outlying datapoints. Four biological replicates were conducted for each strain. The average hyphal formation for all the cells in each frame has been plotted. LEE = Lee's medium, SPI = Spider medium, YPD = Yeast Peptone Dextrose medium.

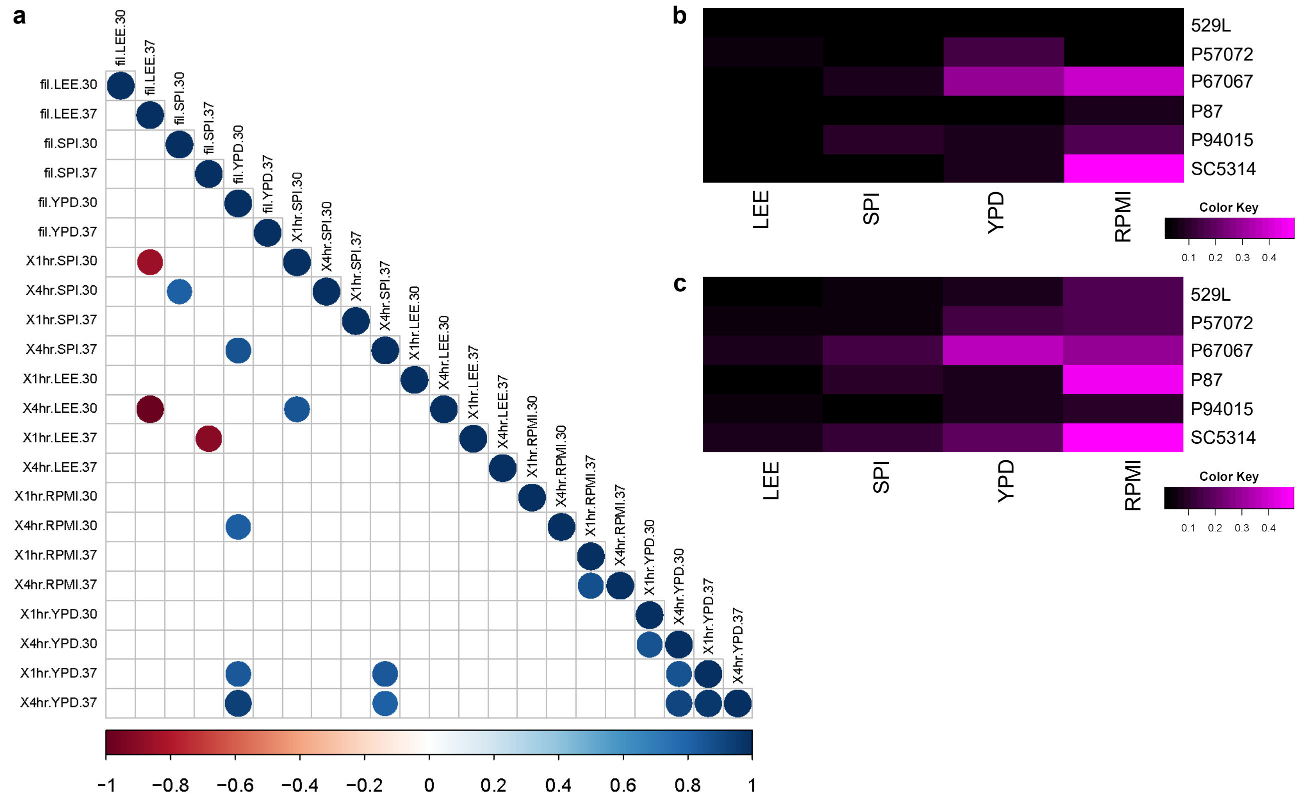

Supplemental Figure 7. *C. albicans* filamentation between solid agar and liquid does not correlate. (**a**) Each filamentation phenotype was correlated to all others by Pearson's correlation. Blue indicates a significant positive correlation and red indicates a significant negative correlation at an adjusted p-value < 0.05. Labels are written as solid/liquid_medium_temperature. "fil" indicates filamentation on solid medium and "X" proceeds the time, in hours, of liquid filamentation. A heatmap of *C. albicans* filamentation in liquid media for four hours at 30°C (**b**) and 37°C (**c**) was constructed from the ratio of filamenting cells per isolate (number of hyphae / number total cells). LEE = Lee's medium, SPI = Spider medium, YPD = Yeast Peptone Dextrose medium. RPMI = RPMI 1640 medium.

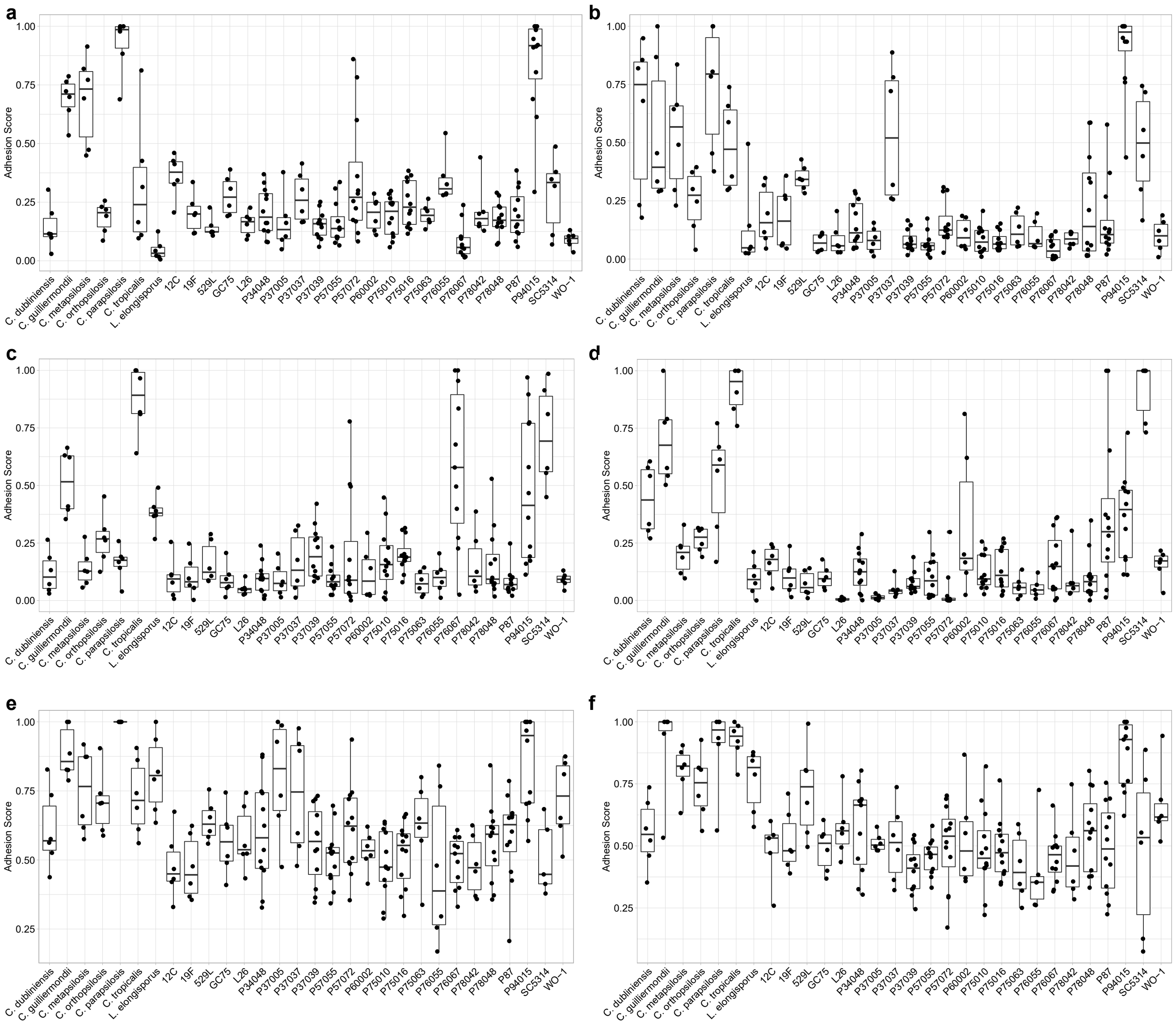

Supplemental Figure 8. Adhesion of Candida isolates. Adhesion data has been plotted for 30°C (LEE (**a**), SPI (**c**), YPD (**e**)) and 37°C (LEE (**b**), SPI (**d**), YPD (**f**)). Overlaid box plots cover the upper and lower interquartile ranges with the intervening line indicating the mean. Whiskers extend +/- two standard deviations. A minimum of six biological replicates were conducted per strain. LEE = Lee's medium, SPI = Spider medium, YPD = Yeast Peptone Dextrose medium.

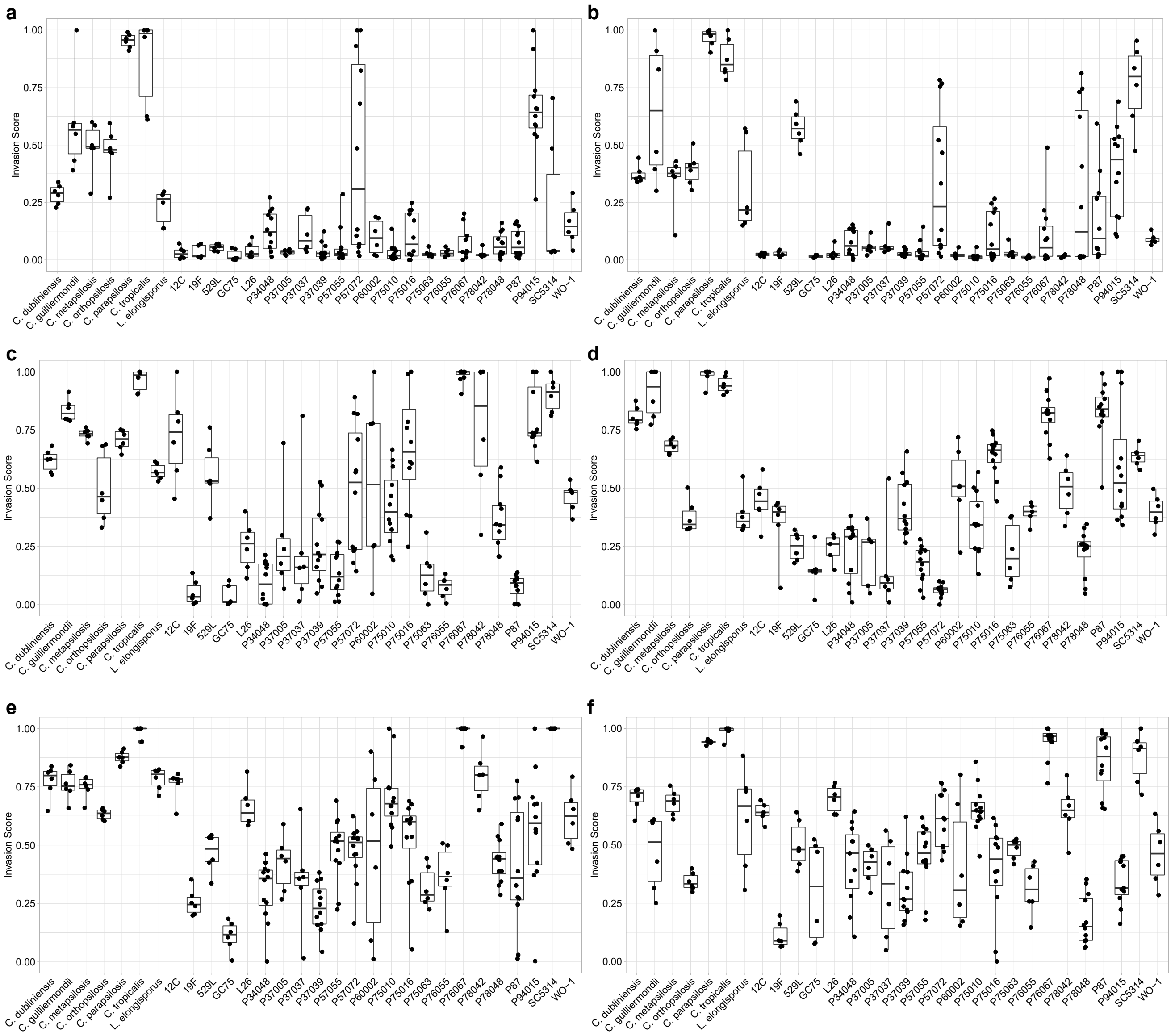

Supplemental Figure 9. *Candida* isolates invasion. Invasion data has been plotted for 30°C (LEE (**a**), SPI (**c**), YPD (**e**)) and 37°C (LEE (**b**), SPI (**d**), YPD (**f**)). Overlaid box plots cover the upper and lower interquartile ranges with the intervening line indicating the mean. Whiskers extend +/- two standard deviations. A minimum of six biological replicates were conducted per strain. LEE = Lee's medium, SPI = Spider medium, YPD = Yeast Peptone Dextrose medium.

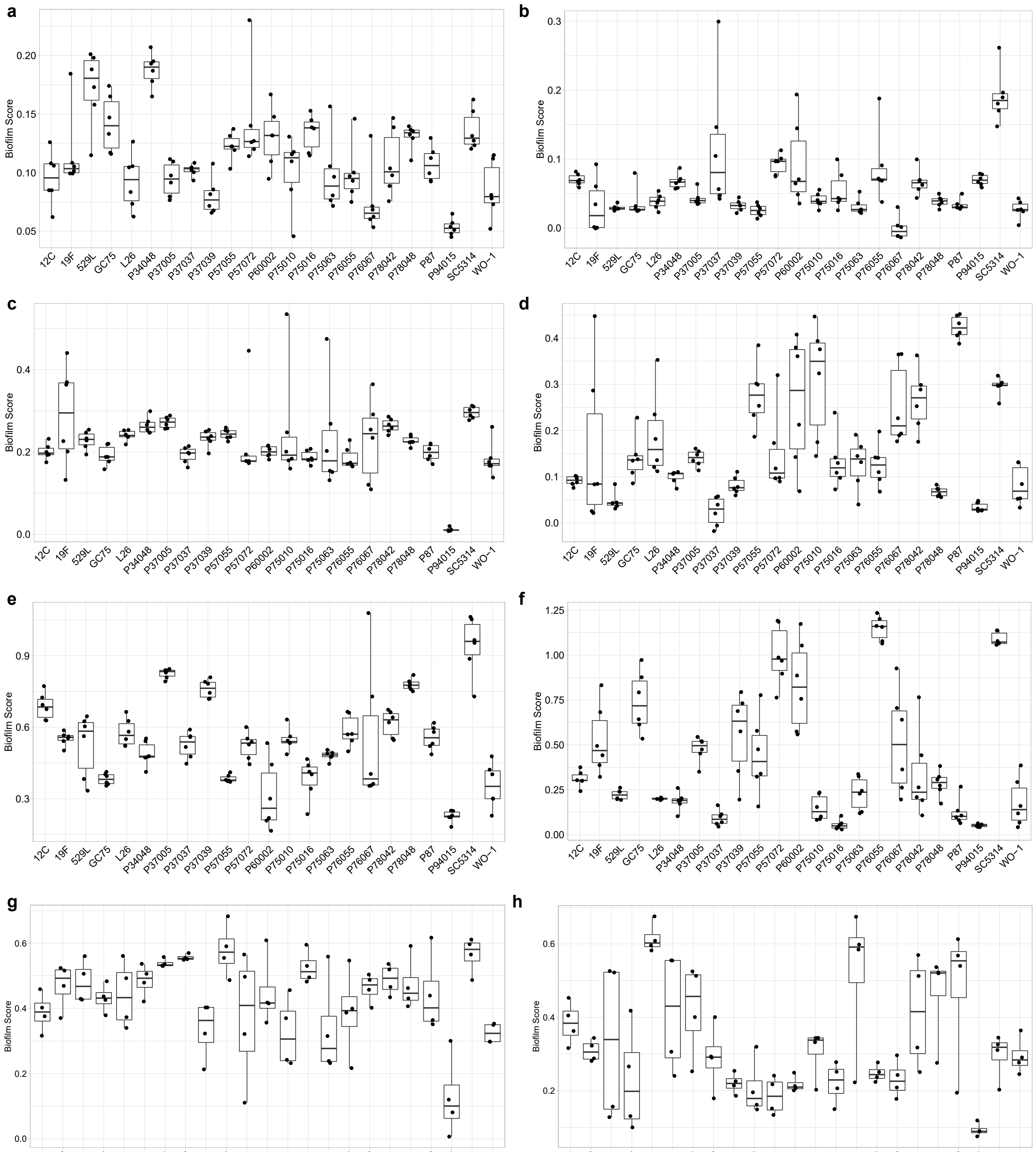

Supplemental Figure 10. *Candida* isolates biofilm formation. Biofilm formation data has been plotted for 30°C (LEE (**a**), SPI (**c**), YPD (**e**), RPMIS (**g**)) and 37°C (LEE (**b**), SPI (**d**), YPD (**f**), RPMIS (**h**)). Overlaid box plots cover the upper and lower interquartile ranges with the intervening line indicating the mean. Whiskers extend +/- two standard deviations. A minimum of six biological replicates were conducted per strain. LEE = Lee's medium, SPI = Spider medium, YPD = Yeast Peptone Dextrose medium. RPMIS = RPMI 1640 supplemented with 10% FBS.

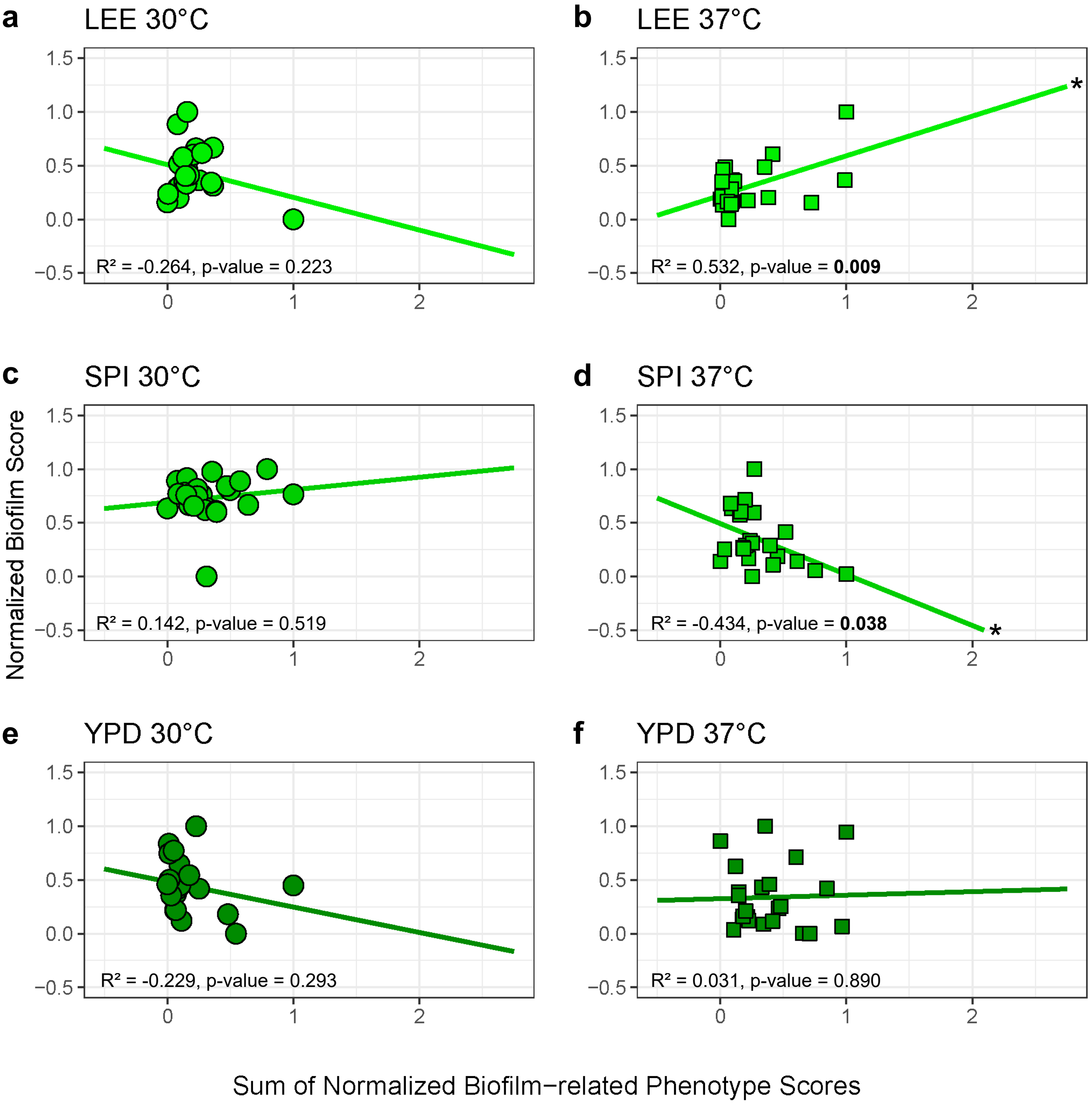

Supplemental Figure 11. Weighted analysis of biofilm phenotypic correlations. Normalized filamentation, adhesion, and invasion scores were weighted for each tested strain as described in Table S2, summed, and plotted (y-axis) against the biofilm score (x-axis) for isolates at 30°C (LEE (**a**), SPI (**c**), YPD (**e**)) and 37°C (LEE (**b**), SPI (**d**), YPD (**f**)).

Assay	Kruskal-Wallis Test			Kolmogorov-Smirnov Test		
**Radial filamentation**	Tested Conditions	KW Statistic	p-value	Tested Conditions	KS Statistic	p-value
	30°C vs. 37°C	0.00330473	0.954157445	(YPD vs. SPI):	0.782608696	3.55E-14
				(YPD vs. LEE):	0.97826087	4.44E-16
				(SPI vs. LEE):	1	4.44E-16
**Colony wrinkling**	Tested Conditions	KW Statistic	p-value	Tested Conditions	KS Statistic	p-value
				(YPD vs. SPI):	0.260869565	0.087259115
				(YPD vs. LEE):	0.913043478	0
				(SPI vs. LEE):	0.891304348	2.22E-16
				(30 vs. 37):	0.420289855	1.02E-05
**Adhesion**	Tested Conditions	KW Statistic	p-value	Tested Conditions	KS Statistic	p-value
	YPD vs. SPI vs. LEE	72.2246671	2.07E-16			
	30°C vs. 37°C	3.89554066	0.048414454			
**Invasion**	Tested Conditions	KW Statistic	p-value	Tested Conditions	KS Statistic	p-value
	30°C vs. 37°C	0.006206	0.937209072	(YPD vs. SPI):	0.347826087	0.007254591
				(YPD vs. LEE):	0.782608696	3.55E-14
				(SPI vs. LEE):	0.652173913	1.54E-09
**Biofilm**	Tested Conditions	KW Statistic	p-value	Tested Conditions	KS Statistic	p-value
	30°C vs. 37°C	12.8708753	0.000333735	(YPD vs. SPI):	0.717391304	1.09E-11
				(YPD vs. LEE):	0.847826087	4.44E-16
				(SPI vs. LEE):	0.630434783	6.86E-09
**Liquid filamentation**	Tested Conditions	KW Statistic	p-value	Tested Conditions	KS Statistic	p-value
	30°C vs. 37°C	5.4289966	0.019805056	(YPD vs. SPI):	0.416666667	0.255775185
				(YPD vs. LEE):	0.666666667	0.007859014
				(SPI vs. LEE):	0.333333333	0.53609777

Supplemental Table 1. Tests for the effect of media and temperature on biofilm phenotypes. LEE = Lee's medium, SPI = Spider medium, YPD = Yeast Peptone Dextrose medium.

Assay	Media	Temperature	Pearson Statistic	Pearson Correlation	P-value
**Radial filamentation**	LEE	30	-0.207243834	-0.152295271	0.837815881
	LEE	37	0.480277033	0.480557643	0.635995473
	SPI	30	-1.496457537	-0.185947529	0.149413543
	SPI	37	0.566624644	0.469851219	0.576975754
	YPD	30	1.283143523	0.245580459	0.213422503
	YPD	37	-1.247796316	0.038348058	0.225840823
**Adhesion**	LEE	30	-1.250162871	-0.263189808	0.224992461
	LEE	37	2.459540854	0.472906948	0.022670473
	SPI	30	-0.516940171	-0.11209464	0.610599802
	SPI	37	2.050119724	0.408369391	0.053044385
	YPD	30	-1.100301825	-0.233469988	0.283654025
	YPD	37	-2.098621363	-0.416371724	0.048122178
**Invasion**	LEE	30	-0.706141724	-0.152295271	0.487860937
	LEE	37	2.511156591	0.480557643	0.020283042
	SPI	30	-0.867243635	-0.185947529	0.395610434
	SPI	37	2.439127107	0.469851219	0.023685008
	YPD	30	1.16094353	0.245580459	0.258692214
	YPD	37	0.175862237	0.038348058	0.862086967
**Adhesion + Radial filamentation**	LEE	30	-1.019550332	-0.21717407	0.319544593
	LEE	37	1.851193921	0.3745567	0.078257719
	SPI	30	-1.069363749	-0.22724898	0.297044039
	SPI	37	1.48087893	0.307497131	0.153487621
	YPD	30	0.076897273	0.016777999	0.939433203
	YPD	37	-2.855240814	-0.528817215	0.009476362
**Adhesion + Invasion**	LEE	30	-1.008602221	-0.214950323	0.324646374
	LEE	37	2.878523233	0.531912849	0.008991147
	SPI	30	-0.761343496	-0.163892283	0.454917297
	SPI	37	2.628571759	0.497559095	0.015700324
	YPD	30	-0.016154816	-0.003525248	0.987263384
	YPD	37	-1.239824357	-0.261162326	0.228716631
**Radial filamentation + Invasion**	LEE	30	-0.718953443	-0.154992605	0.480093975
	LEE	37	1.933467341	0.388733484	0.066772517
	SPI	30	-1.249736446	-0.263106251	0.225145145
	SPI	37	1.830462532	0.370942054	0.081411008
	YPD	30	1.373559224	0.287115166	0.184065757
	YPD	37	-0.671956919	-0.145081599	0.508936979
**Adhesion + Invasion + Radial filamentation**	LEE	30	-1.068600492	-0.22709515	0.297380019
	LEE	37	2.52329343	0.482340893	0.019756854
	SPI	30	-1.051465439	-0.223637202	0.304994526
	SPI	37	2.142625535	0.423549302	0.044016475
	YPD	30	0.708750324	0.152844755	0.486273626
	YPD	37	-1.942806744	-0.390325721	0.065567622

Supplemental Table 2. Correlations of biofilm-related phenotypes to biofilm mass. LEE = Lee's medium, SPI = Spider medium, YPD = Yeast Peptone Dextrose medium.

Condition	Log2(Weighting Factor)
adh_LEE_30	4
adh_LEE_37	2
adh_YPD_30	-4
adh_YPD_37	2
adh_SPI_30	-4
adh_SPI_37	2
fil_LEE_30	-1
fil_LEE_37	-4
fil_YPD_30	4
fil_YPD_37	0
fil_SPI_30	4
fil_SPI_37	-4
inv_LEE_30	-1
inv_LEE_37	2
inv_YPD_30	4
inv_YPD_37	-4
inv_SPI_30	-2
inv_SPI_37	3

Supplemental Table 3. Phenotypic weighting factors for additive contributions to biofilm formation. Written as phenotype_medium_temperature. Adh indicates adhesion, fil indicates filamentation, and inv indicates invasion. LEE = Lee's medium, SPI = Spider medium, YPD = Yeast Peptone Dextrose medium.

Species	Identifier	Body site of isolation	MLST fingerprinting clade	Reference
Candida albicans	GC75	Oral cavity	SA	Hirakawa et al. 2015.
Candida albicans	P75016	Bloodstream	SA	Hirakawa et al. 2015.
Candida albicans	P75063	Bloodstream	SA	Hirakawa et al. 2015.
Candida albicans	P87	Oral cavity	SA	Hirakawa et al. 2015.
Candida albicans	P60002	Bloodstream	SA	Hirakawa et al. 2015.
Candida albicans	P94015	Bloodstream	I	Hirakawa et al. 2015.
Candida albicans	P34048	Bloodstream	III	Hirakawa et al. 2015.
Candida albicans	P78042	Bloodstream	III	Hirakawa et al. 2015.
Candida albicans	P57055	Bloodstream	III	Hirakawa et al. 2015.
Candida albicans	P57072	Bloodstream	II	Hirakawa et al. 2015.
Candida albicans	P76055	Bloodstream	II	Hirakawa et al. 2015.
Candida albicans	P76067	Bloodstream	II	Hirakawa et al. 2015.
Candida albicans	P75010	Bloodstream	E	Hirakawa et al. 2015.
Candida albicans	19F	Vagina	I	Hirakawa et al. 2015.
Candida albicans	L26	Vagina	I	Hirakawa et al. 2015.
Candida albicans	P37039	Bloodstream	I	Hirakawa et al. 2015.
Candida albicans	12C	Oral cavity	I	Hirakawa et al. 2015.
Candida albicans	P37005	Oral cavity	I	Hirakawa et al. 2015.
Candida albicans	P37037	Oral cavity	I	Hirakawa et al. 2015.
Candida albicans	P78048	Bloodstream	I	Hirakawa et al. 2015.
Candida albicans	SC5314	Bloodstream	I	Hirakawa et al. 2015.
Candida albicans	529L	Oral cavity	Unassigned	Cuomo et al. 2019.
Candida albicans	WO-1	Bloodstream	Unassigned	Butler et al. 2010.
Candida dubliniensis	CD36	Oral cavity	Unassigned	Butler et al. 2010.
Candida tropicalis	MYA3404	Unknown	Unassigned	Butler et al. 2010.
Candida parapsilosis	J941367	Unknown	Unassigned	Butler et al. 2010.
Candida metapsilosis	ATCC96143	Unknown	Unassigned	Butler et al. 2010.
Candida orthopsilosis	90-125	Unknown	Unassigned	Butler et al. 2010.
Lodderomyces elongisporus	YB-4239	Unknown	Unassigned	Butler et al. 2010.
Candida guillermondii	ATCC6260	Unknown	Unassigned	Butler et al. 2010.

Supplemental Table 4. Strains used in this study.

SUPPLEMENTAL DATA SETS

Supplemental Data set 1. This Supplemental Data set includes the recipe script file (.rcp) for use with the MIPAR software package (www.mipar.us) to detect radial filamentation on Lee's medium.

Supplemental Data set 2. This Supplemental Data set includes the recipe script file (.rcp) for use with the MIPAR software package (www.mipar.us) to detect radial filamentation on Spider medium.

Supplemental Data set 3. This Supplemental Data set includes the recipe script file (.rcp) for use with the MIPAR software package (www.mipar.us) to detect radial filamentation on YPD medium.

Supplemental Data set 4. This Supplemental Data set includes the recipe script file (.rcp) for use with the MIPAR software package (www.mipar.us) to detect agar adhesion.

Supplemental Data set 5. This Supplemental Data set includes the recipe script file (.rcp) for use with the MIPAR software package (www.mipar.us) to detect agar invasion.

Supplemental Data set 6. This Supplemental Data set includes the recipe script file (.rcp) for use with the MIPAR software package (www.mipar.us) to detect liquid filamentation.

SUPPLEMENTAL METHODS

The following detection protocols, accessible here, reference functions detailed in the online manual for MIPAR (https://www.manula.com/manuals/mipar/user-manual/latest/en/topic/getting-started). Detection parameters are described below and include guidelines for modifying steps based on the condition of the user's starting images. While these protocols have been adapted for imaging within a BioRad ChemiDoc XRS+ system, all of the steps detailed below may be modified to meet different image inputs and desired measurement parameters.

Plate detection for solid agar assays

Images taken through plate-based assays were processed by the visual analysis tool MIPAR, version 3.0.3 (MIPAR, Worthington, OH). Images were acquired using a ChemiDoc XRS+ imager from the top of the plate. While this imaging platform provided easily reproducible imaging conditions, any imaging platform which allows for the top-down imaging of a plate surface may be used. The most important factor affecting these imaging conditions is light source. In the ChemiDoc XRS+ imager, two light sources angled from above and either side of the camera reduced colony shadow effects and allowed for consistent lighting. Most image types, including proprietary formats, can be read through the MIPAR software. The images collected here were originally in BioRad proprietary format before being exported as .tif image files for processing.

This outline comprises a series of steps to analyze the image and output a radial filamentation score, agar adhesion score, and agar invasion score in which each set of detection parameters have been highlighted for each phenotypic category as labeled in

the detection recipe. Depending on image format and processing, inversion of the image colors may be necessary to convert the image back to its original contrast after uploading it to the software. To remove bright spots caused by lights during imaging, the area of interest was standardized to a maximal brightness where the bright spots were loaded in as a companion image. Grayscale reconstruction was used to standardize the center colony brightness across the colony surface. To limit the area of interest to the imaged plate, the full plate was selected from the black background. The edges of the plate were then removed from analysis to omit colonies which contact the edge of the plate. Dilation, filling, and erosion steps were added to select colonies away from the plate background through detecting differences in coloration. As media types are differentially pigmented, these steps will need to be adjusted to the type of plate and plastic thickness since shadowing from the light source may cause errors. This detection was saved as a companion image (a reference image which can later be used to overlay and select area within another detection) to select colony material without edge obstruction. Each published recipe contains modifications to this step based on media darkness.

Solid plate filamentation detection

The MIPAR recipes [LEE_radial.rcp, SPI_radial.rcp, and YPD_radial.rcp] were used to detect filamentation on Lee's, Spider, and YPD media plates. The following adapting thresholding steps are entirely dependent on media coloration, transparency, and imaging background. As such, adjustments here should focus on ensuring individual hyphae can be appropriately detected. To detect separate colonies and, in particular, radial filamentation, an adaptive threshold based on pixel brightness using a Gaussian

mean statistic was selected. An intersection operation was conducted using the previously defined companion image to detect filaments within the mask of the colonies on the plate which did not touch the edge. A series of separate feature steps, to separate defined colonies from one another, and a retain dilation step, to ensure capture of all filament pixels, were used to collect all undetected filaments such as those that appear discontinuous around the perimeter of the colony or may be intertwined into thicker bundles (therefore possessing brightness closer to the center colony). These steps also rejected erroneously detected elements (such as a scrape on the agar or a chip on the plate surface). This step defines the total radial area of the filaments. Two potential modifications were tested and may be applicable to different data sets. First, simple detection utilizing brightness was mostly successful at detecting radial filaments but was less accurate when colonies formed thick bundles of radial filaments (such as for strain P87). If imaged colonies do not display this phenotype, a simplified detection utilizing a smart clustering approach may be sufficient for detecting radial filaments. Second, higher colony densities require alteration of current and additional thresholding to tease apart filamentation from colonies whose filaments overlap. Due to the general uniformity of center colony structure in this dataset, detection of the center colony can be done simply by adjusting the brightness. Smart clustering, following by range thresholding, defined the brighter colony center structures from radial hyphal projections. A smoothing and separate features step separated individual colonies from one another before final tallying of colonies which were not in contact with the plate edge. Final radial filamentation was measured as $(\text{area}_{\text{hyphal growth}} - \text{area}_{\text{center colonies}}) / (\text{area}_{\text{center colonies}})$. The steps in this

protocol are labeled by category for ease of usage and modification as well as coordinated with memory images which can be used further for additional detection or assessment.

Solid plate adhesion detection

The MIPAR recipe [Adhesion_detection.rcp] was used for detection and quantification of plate-based adhesion. Briefly, the plate area was detected (described in detail above), and bright spots were removed. Colonies were then isolated by applying the separate features process. After colony detection and separation, the pixels occupied by colonies were counted. In order to determine total adhesion, adhesion detection was applied to both the original image (prior to wash) and the washed image. Adhesion was scored as $(area_{colonies\ post\text{-}wash}) / (area_{colonies\ pre\text{-}wash})$.

Solid plate invasion detection

Agar invasion followed the same initial plate detection and continued with the MIPAR recipe [Invasion_detection.rcp]. After the plate area was selected, a simple detection was conducted to select the remaining invasion profile from the darker plate background. Smart clustering separated the image area into contrast bins and was followed by a basic threshold to isolate invasion profiles from the agar. Steps were included to eliminate both over detection (occurring when there was no invasion profile to be detected) as well as eliminate erroneous detection of individual pixels that did not match the plate background. Invasion detection was then applied to both the original image (prior to treatments) and the image of the plate following direct wiping of all colony material from the plate, scored as $(area_{colonies\ agar\ invasion}) / (area_{colonies\ pre\text{-}wash})$.

Liquid filamentation detection

Images of fungal cells grown in liquid media and prepared on glass slides were processed by the visual analysis tool MIPAR, version 3.0.3 (MIPAR, Worthington, OH). The liquid detection recipe [Liquid_detection.rcp] was used to determine filamentation scores from images collected across four liquid media types: Lee's, Spider, YPD, and RPMI. This liquid detection recipe used a series of steps to select cells and bin them into either "yeast" or "hyphae" categories, providing precise morphology counts for each image. The first four steps of the recipe separated cells from the background: Step 1 produced a histogram equalization in 10-pixel blocks, equalizing the brightness and contrast across each block of the image. Step 2 applied a Wiener filter to the image, which reduced noise and set each pixel equal to the median pixel value among 19-pixel blocks in order to best normalize the background of the image. This step may require adjustment depending on the light source, background debris, and resolution of the image. Step 3 "flattened" the image background by applying a Gaussian blur to the image and subtracting the blur from the reference image. Finally, a non-local means filtering algorithm was applied to the image, which compares neighboring windows of pixels to each other and adjusts the windows' medians to reduce noise and sharpen differences in the image. The combination of these filtering steps created images where cells appear very dark against a light grey background. The parameters in these steps may need to be adjusted depending on the type of microscope used to acquire images and/or microscope settings used. The filtering steps produced a black and white image, where the cells appeared black against a white background. The rest of the recipe dictates automated detection of cells in the image. This segment of the recipe filled all holes within detected cells and rejected any selected items outside of the 250-50000-pixel range to

eliminate any debris in the image that was incorrectly detected. A memory image was set with all the remaining cells selected. Finally, any cell with a roundness score less than 0.43 were designated true hyphae, while cells with a roundness greater than or equal to 0.43 were binned as yeast. The final filamentation score was calculated as total hyphal count across all images of a biological replicate divided by the total cells across all images of the replicate.

Weighted Composite Phenotype Scores to Biofilm Scores Correlations.

Weighting was determined by identifying the $\log_2$ multiplier that produced the strongest association of each individual phenotype to total biofilm formation by Pearson correlation. This multiplier was applied to each corresponding biofilm-related phenotype and summed as a composite score. The composite score was then plotted against biofilm formation for the *C. albicans* isolates. The code for this calculation is in the OSU git repository: https://code.osu.edu/fillinger.22/mipar_fwaibl-r2r-weighted_phenotypes
